# Mix and Match Tuning of the Conformational and Multistate Redox Switching Properties of an Overcrowded Alkene

**DOI:** 10.1021/jacs.4c08284

**Published:** 2024-09-13

**Authors:** Robert Hein, Charlotte N. Stindt, Ben L. Feringa

**Affiliations:** Stratingh Institute for Chemistry, University of Groningen, Nijenborgh 4, 9747AG Groningen, The Netherlands

## Abstract

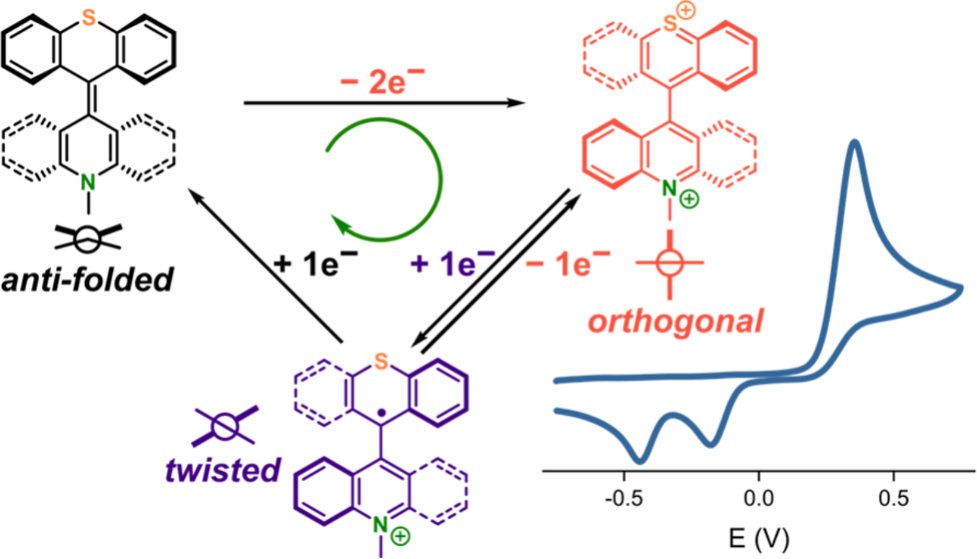

Overcrowded alkenes have received considerable attention as versatile structural motifs in a range of optical switches and light-driven unidirectional motors. In contrast, their actuation by electrochemical stimuli remains underexplored, even though this alternative energy input may be preferred in various applications and enables additional control over molecular switching states and properties. While symmetric bistricyclic overcrowded enes (BAEs) containing two identical halves based on either thioxanthene (TX) or acridine (Acr) motifs are known to be reversible conformational redox switches, their redox potentials are generally too high or low, respectively, thereby preventing wider applications. Herein, we demonstrate that the “mixed” TX-Acr switch possesses redox properties that lie between those of its parent symmetric analogs, enabling interconversion between three stable redox and conformational states at mild potentials. This includes the neutral *anti-*folded, the dicationic orthogonal, and a unique twisted monoradical cation state, the latter of which is only accessible in the case of the mixed TX-Acr switch and in a pathway-dependent manner. Consequently, with this multistate redox switch, a myriad of molecular properties, including geometry, polarity, absorbance, and fluorescence, can be modulated with high fidelity and reversibility between three distinct stable states. More generally, this study highlights the versatility of the “mix and match” approach in rationally designing redox switches with specific (redox) properties, which in turn is expected to enable a myriad of applications ranging from molecular logic and memory to actuators and energy storage systems.

## Introduction

Light-driven molecular switches and motors^1−4^ play a major role in the transition to responsive materials and dynamic molecular systems with a myriad of applications, ranging from molecular machinery^5−9^ to photopharmacology^10−13^ and smart materials.^14−19^ This is also reflected in a variety of distinct photoswitches with diverse chemical structures and properties.^20−22^ In contrast, the development of small-molecule redox-driven conformational switches remains relatively underexplored^3,23^ compared to the eminent examples comprising large, complex (interlocked) molecules in which redox switching gives rise to changes in intercomponent noncovalent interactions and associated dynamic changes.^24−29^ The exploration of small-molecule redox switches undergoing well-defined and highly reversible structural changes remains an important challenge. Notably, the so-called dynamic redox (DYREX)^a^ systems based on reversible C–C single bond or C=C double bond formation/breaking (and associated geometric changes) have been studied as, for example, electrochromic switches.^30−34^ In addition, these DYREX systems have potential in molecular memory and computing,^34,35^ energy storage,^36^ molecular actuators,^37^ and in fundamental studies of aromaticity, molecular conformations, and organic radicals.^38−42^ However, this highly promising application scope remains largely unrealized, which can in part be attributed to the relative lack of DYREX switches with favorable redox properties and/or their limited stability and, as a consequence, not allowing multiple switching cycles without fatigue.

**Figure 1:**
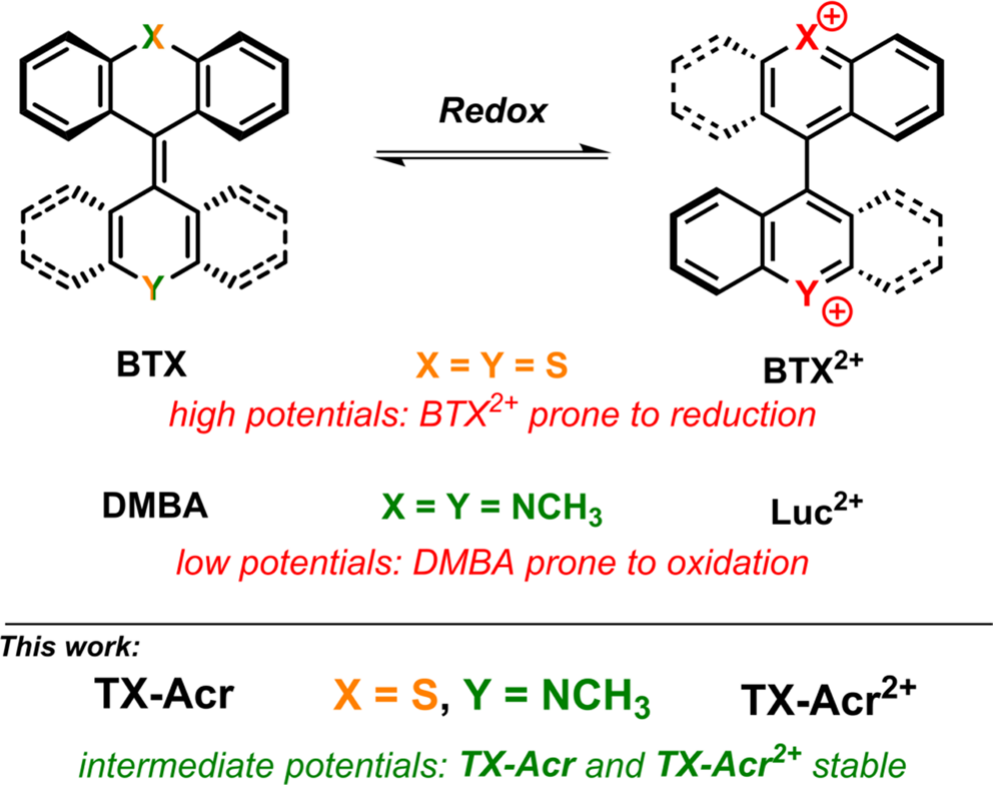
Overview of chemical structures of redox-active bistricyclic aromatic enes (BAEs) including the symmetric **BTX** and dimethylbiacridylene (**DMBA**) as well as the “mixed” **TX-Acr**.

A noteworthy DYREX switch scaffold we introduced, which displays a very high degree of chemical stability in all switching states, is bisthioxanthylidene (**BTX**, Figure 1 where X = Y = S), an overcrowded bistricyclic aromatic ene (BAE) with rich optical, stereochemical, conformational, and redox switching properties.^39,40,43,44^ In its neutral state, **BTX** adopts, due to significant steric crowding in the vicinity of the central alkene bond, an *anti*-folded conformation, which, upon two-electron oxidation to the dicationic **BTX**^**2+**^, is converted to an orthogonal species containing a central single bond. This is associated with a significant rotation of the respective halves of the switch and furthermore results in large changes in the polarity, absorbance, and fluorescence.^39^ This switching process is highly reversible, as both neutral and dicationic states are chemically stable and isolable. However, the potential for the simultaneous two-electron oxidation from **BTX** to **BTX**^**2+**^ is very high (≈1.2 V vs saturated calomel electrode (SCE)), restricting its further exploration in various applications, especially in media in which other functional groups, solvents, or electrolytes with low oxidation potential are present.^39,40^ For example, this oxidation potential is, depending on exact conditions and pH, close to that of the oxidation of water or halides, thereby hampering switching in aqueous electrolytes.

Similarly, the dinitrogen analog of this overcrowded alkene dimethylbiacridylene (**DMBA**, X = Y = NCH_3_, Figure 1) undergoes the same redox-induced transformations but suffers from the “opposite” problem; its low redox potentials render the neutral overcrowded alkene state very sensitive to (air) oxidation.^45−47^ In fact, for this structure, the dicationic lucigenin (**Luc**^**2+**^) state is the more stable form and is, as nitrate salt, often employed as a commercially available chemoluminescent probe.^48,49^

Conceivably, the combination of both a nitrogen- and a sulfur-containing rotor half in a mixed BAE switch would induce intermediate redox properties such that both neutral and dicationic states are stable and can be interconverted at more accessible potentials. Herein, we show that the mixed thioxanthylidene-acridene (**TX-Acr**, X = S, Y = NCH_3_) indeed displays rich switching properties with high fidelity (reversibility/stability) that generally lie in between those of its symmetric parent congeners while we discovered that, at the same time, it enables a unique pathway-dependent access to a third monoradical cation redox state.

## Results and Discussion

### Synthesis and Characterization

**Scheme 1:**
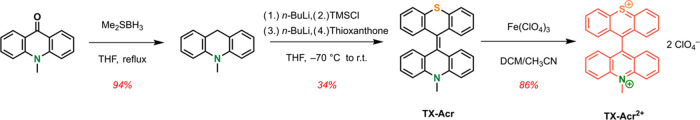
Synthesis of **TX-Acr**

The mixed, heteromerous^50^ overcrowded alkene **TX-Acr** was obtained in two steps via an adapted Peterson olefination,^51^ as shown in Scheme 1. Commercially available *N*-methylacridin-9(10*H*)-one was first reduced to *N*-methyl-9,10-dihydroacridine,^52^ which was subsequently silylated by reaction with *n-*BuLi and TMSCl in tetrahydrofuran (THF). Further deprotonation with *n-*BuLi generated the corresponding α-silyl carbanion, which was reacted with thioxanthen-9-one to afford the yellow, strongly fluorescent **TX-Acr** in moderate yield. Alternatively, the title compound can also be synthesized via palladium-catalyzed annulation of phenazastannines.^53^

Chemical oxidation of **TX-Acr** with Fe(ClO_4_)_3_ induced clean conversion to the dication **TX-Acr**^**2+**^, which was isolated as an orange-red bench-stable solid in high yield. All compounds were fully characterized by ^1^H and ^13^C NMR as well as high-resolution mass spectrometry (HRMS) as shown in Figures S1–S4.

**Figure 2:**
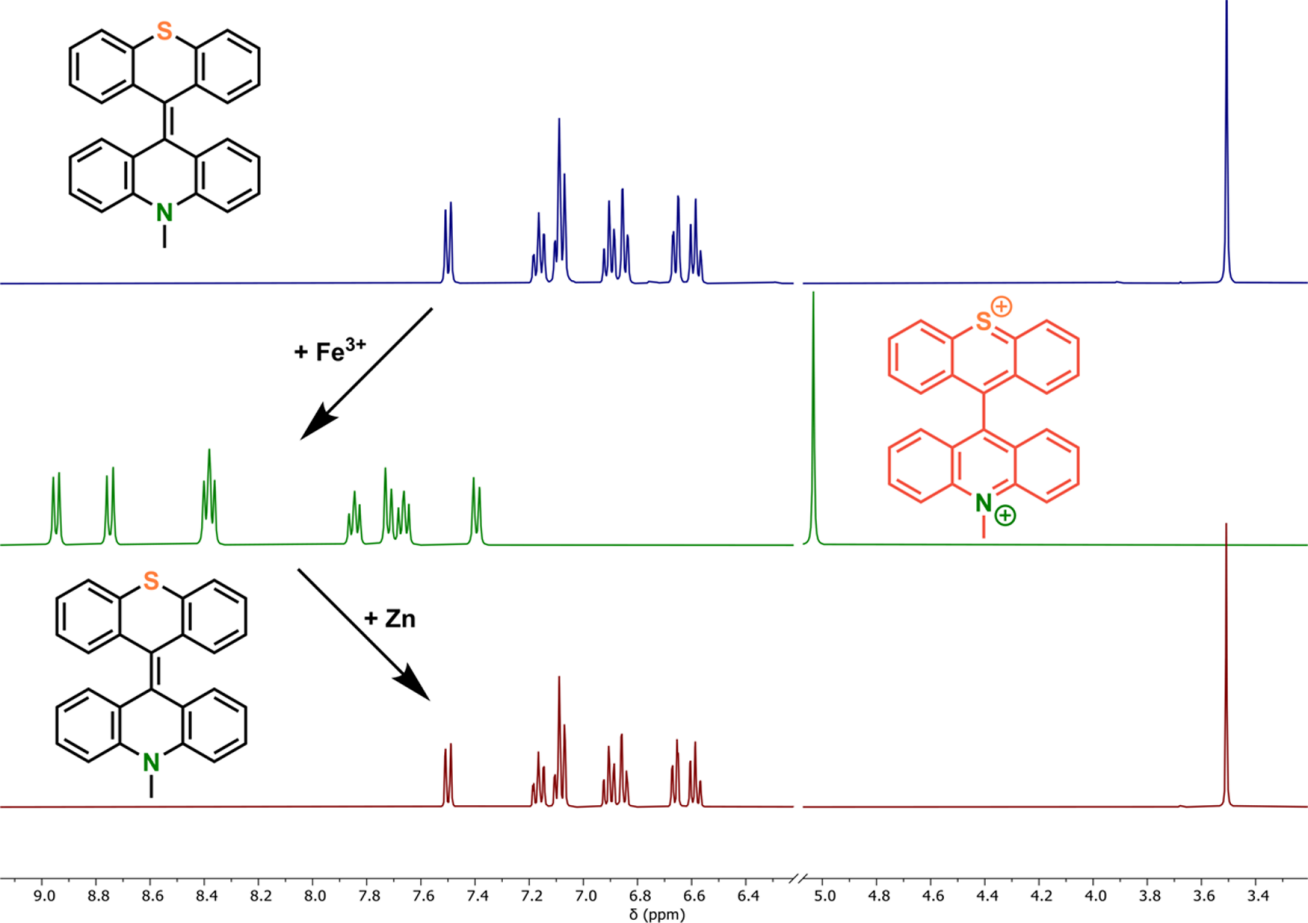
Stacked ^1^H NMR spectra of **TX-Acr** (top), **TX-Acr**^**2+**^ (middle), and rereduced **TX-Acr** (bottom) in 1:1 CD_2_Cl_2_/CD_3_CN.

The ^1^H NMR spectrum of **TX-Acr**^**2+**^ shows the same number of distinct protons as that of the neutral **TX-Acr**, all of which are downfield shifted in comparison to the neutral parent compound (Figure 2), confirming a symmetric, dicationic, closed-shell structure. Importantly, chemical reduction with zinc cleanly afforded the neutral parent compound again, confirming the high reversibility of the redox interconversion of this switch (Figure 2, bottom).

### Conformational Analysis

**Figure 3:**
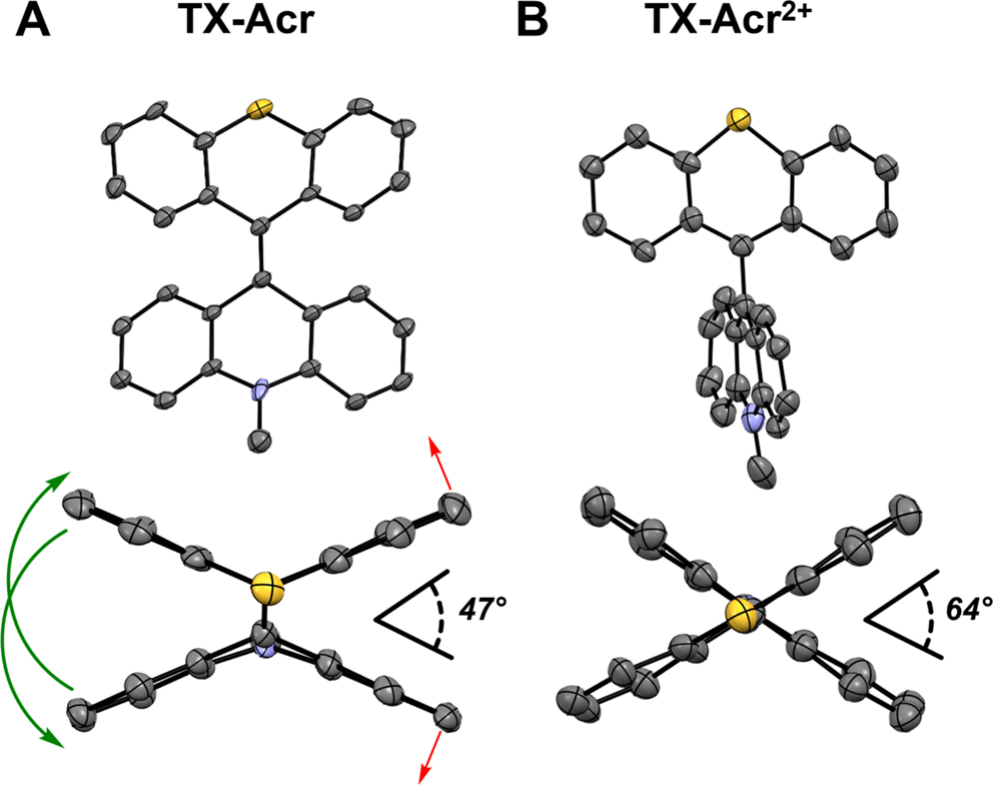
Different ORETEP image views of (A) **TX-Acr** and (B) **TX-Acr**^**2+**^**(ClO**_**4**_^**–**^**)**_**2**_. Ellipsoids are drawn at 50% probability, and hydrogens and counteranions have been omitted for clarity. The green and red arrows depict the rotation of each of the two sides of the switch upon oxidation of **TX-Acr** to **TX-Acr**^**2+**^**(ClO**_**4**_^**–**^**)**_**2**_. The angles were defined as the angles between the mean planes of the rotors. For **TX-Acr**, these planes were defined by one phenyl group on each switch half (on the same side of the double bond). For **TX-Acr**^**2+**^**(ClO**_**4**_^**–**^**)**_**2**_, the planes were defined by both phenyl rings of each rotor.

To investigate the conformational properties of the switch, single-crystal X-ray diffraction (XRD) studies were carried out. Upon layering toluene on top of a solution of **TX-Acr** in dichloromethane (DCM), yellow single crystals were obtained, and their structure confirmed the expected *anti-*folded conformation of the neutral switch state (Figure 3A), as also observed for both parent compounds **BTX**(54) and **DMBA**.^55^ Specifically, the two rotor halves adopt “V” shapes that fold away from each other at an angle of 47° between the aromatic motifs on opposite sides of the central fjord region, with a central C=C bond length of 1.359(6) Å. In contrast, the crystal structure of **TX-Acr**^**2+**^**(ClO**_**4**_^**–**^**)**_**2**_, for which single crystals were obtained by slow diffusion of pentane into a solution of the dication in CH_3_CN, revealed almost fully flat rotor halves that adopt a highly twisted (tw), almost perpendicular, arrangement with a 64° angle between the rotor planes in the solid state (Figure 3B). As a result, interconversion of the neutral **TX-Acr** to the dicationic **TX-Acr**^**2+**^ is associated with the significant motion of the switch halves, specifically rotation whereby one side “opens up” by at least 20° (Figure 3A, red arrows), while even larger rotation, and edge-passage, of the other side is observed (≥111°, green arrows). In addition, the central C–C bond, now a single bond, elongates to 1.506(10) Å. These observations are again consistent with expectations and the orthogonal structure of **BTX**^**2+**^ and **Luc**^**2+**^.^39^ It is important to note here that in both of these parent homomerous dications, both rotors adopt a near-perfectly perpendicular geometry, which would also be expected for **TX-Acr**^**2+**^. The small deviations from this “ideal” orthogonality observed here are mostly likely a result of a more favorable crystal packing, as well as the fact that rotation around the central C–C single bond is unrestricted, such that twisted/orthogonal states with angles close to 90° are very close in energy and can interconvert without significant barriers. Indeed, computational studies at the r^2^SCAN-3c level of theory confirm that the lowest-energy conformer of the dication is the fully orthogonal state (see Table S9 and Figures S10–S13). For simplicity, we will in the following refer to the structure of **TX-Acr**^**2+**^ as an orthogonal state.

Similarly, these calculations reproduced the *anti-*folded structure of neutral **TX-Acr** very well (Figure S10) and corroborated that this is indeed the most stable conformer; the *syn-*folded and twisted conformers of **TX-Acr** are calculated to be 39 and 52 kJ/mol higher in energy, respectively (Table S9 and Figure S11). However, the former metastable *syn*-folded state can be populated by irradiation as discussed in the following section.

### Photoswitching Studies

**Figure 4:**
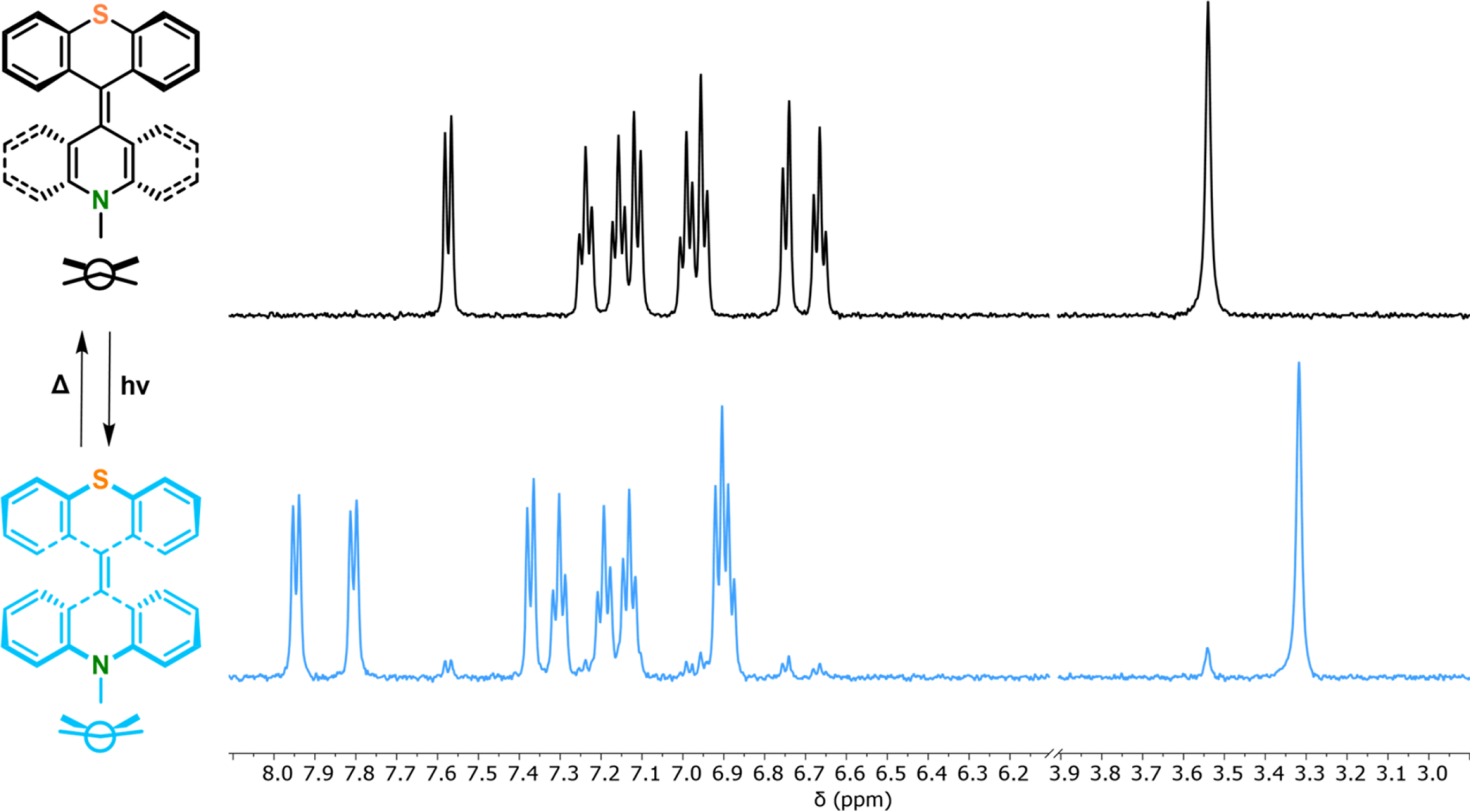
Stacked ^1^H NMR spectra of **TX-Acr** showing the photochemical and thermal interconversions between the *anti-*folded state at 25 °C (top) and the *syn*-folded state obtained by continuous in situ irradiation with 395 nm light in CD_2_Cl_2_ at ∼−90 °C (bottom).

Unlike the vast majority of DYREX systems based on reversible C–C single bond switching, the central C=C double bond of DYREX systems based on overcrowded alkenes is also inherently light-responsive. This includes photochemical *E*–*Z* isomerization as well as interconversion between different folded states. Specifically, both **BTX** and **DMBA** (Figure 1) can be converted from their stable *anti-*folded conformations to the metastable *syn-*folded states by irradiation. The reverse process cannot be driven photochemically but involves purely thermal relaxation. As such, quantitative photoswitching to the *syn-*folded state can be achieved in both cases, albeit at significantly different temperatures. For **BTX**, the energy barrier for the relaxation is sufficiently high to obtain a quantitative population of the *syn-*folded state near room temperature. Previously, a relaxation half-life time of *t*_1/2_ = 22 min at −5 °C was reported, corresponding to an activation barrier of ∼82 kJ/mol.^56^ In contrast, **DMBA**_***syn***_ relaxes rapidly even at −50 °C, at which a relaxation half-life of *t*_1/2_ = 15 s was observed,^56^ corresponding to an activation barrier of ∼60 kJ/mol. To investigate the photoswitching of **TX-Acr**, in situ NMR irradiation studies were carried out at low temperatures. As shown in Figures 4 and S15–S17, irradiation with 395 nm light at ∼−90 °C in CD_2_Cl_2_ induced near quantitative switching to a symmetric species with the same number of distinct protons, which we attribute to the *syn-*folded state. This was corroborated by comparison with the NMR spectra of **DMBA**_***syn***_ and **BTX**_***syn***_, which display the same shift patterns upon switching,^39,56^ as well as NOESY NMR studies (see Figure S18 and associated discussion). Upon thermal relaxation at the same temperature, quantitative conversion back to the *anti-*folded state was observed with a half-life of 15.5 min (Figures S19 and S20). This corresponds to an energy barrier of ∼55 kJ/mol.^56^ Interestingly, this barrier is thus slightly lower than that for **DMBA**. Density functional theory (DFT) calculations confirm this trend (Table S10) and reveal that the low barrier of **TX-Acr** is not a result of ground-state destabilization but rather a result of relative stabilization of the transition state connecting the *syn*- and *anti*-folded conformations, as the mixed structure allows the TX half to adopt its preferred folded geometry while the Acr half is planarized (see Figure S14 and associated discussions for more details).

### Electrochemistry

**Figure 5:**
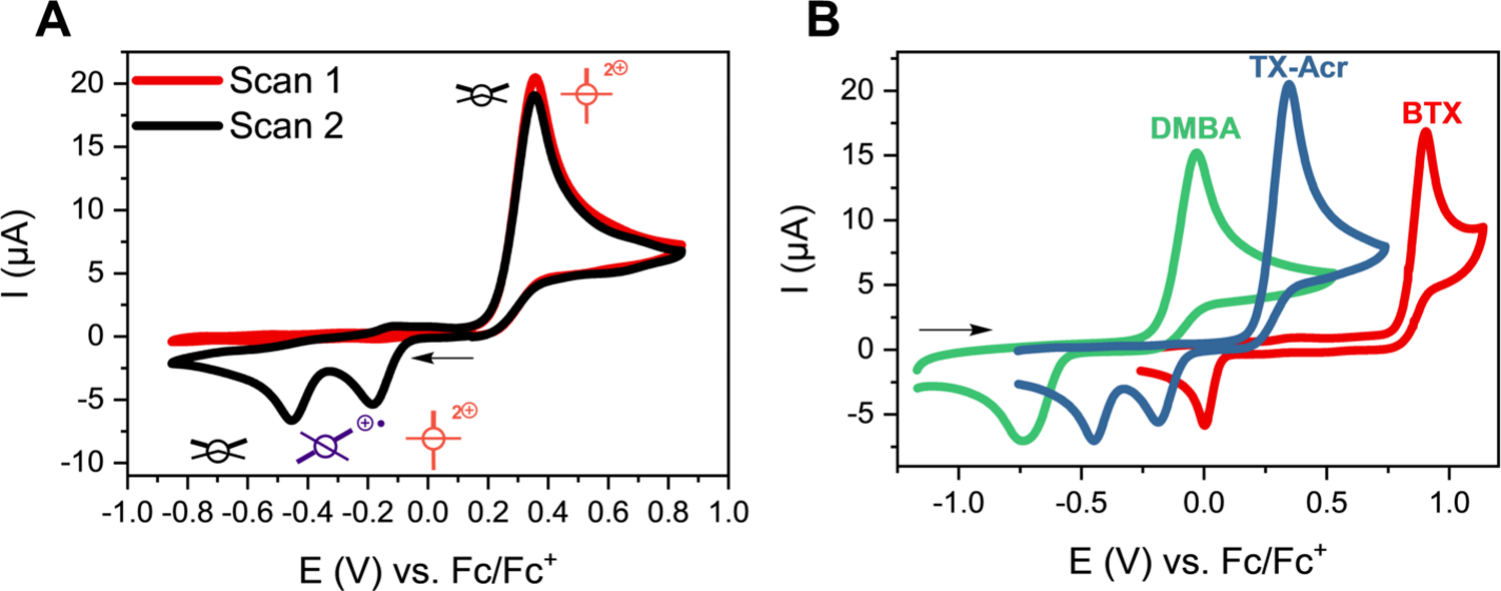
CVs of neutral overcrowded BAEs (0.5 mM) in DCM, 100 mM TBAPF_6_ at a scan rate of ν = 0.1 V/s using a glassy carbon (GC) working electrode. The black arrow indicates the starting point and the initial direction of the first scan. (A) **TX-Acr** with a schematic depiction of the geometric changes associated with each redox wave, see also Figure 6A. On the first scan (red), no reduction peaks were observed as no **TX-Acr**^**2+**^ was generated yet within the chosen potential range. (B) Comparison of symmetric **DMBA** (green) and **BTX** (blue) with mixed **TX-Acr** (red).

**Figure 6:**
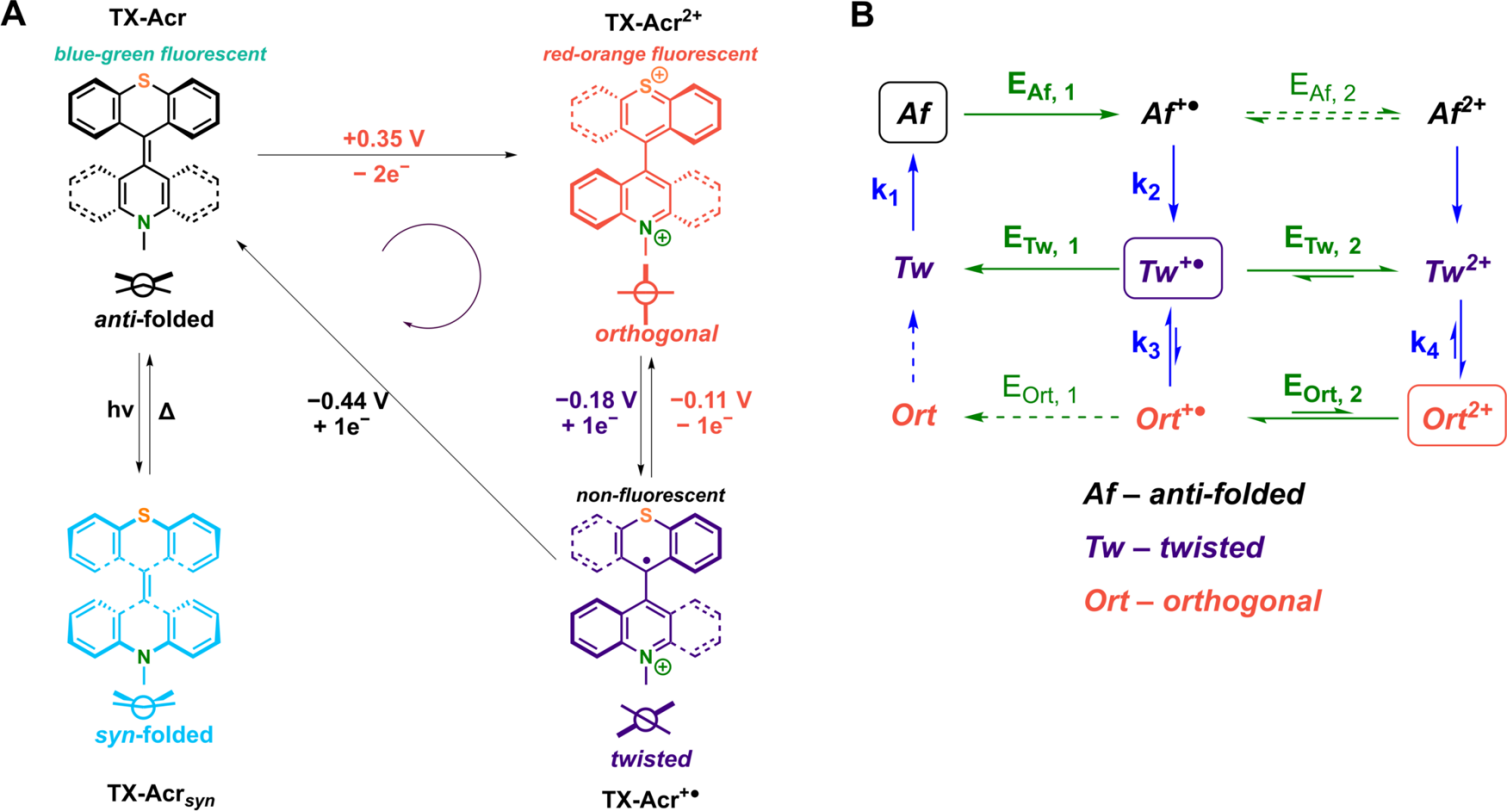
(A) Overview of the four accessible states of **TX-Acr**, their conformations, and their interconversion pathways. The potentials refer to the peak potentials of the respective waves in DCM, 100 mM TBAPF_6_ (vs Fc/Fc^+^). (B) Detailed square scheme depicting the relevant redox (green arrows) and structural (blue arrows) interconversions between all redox states. The major pathways are shown in full/bold arrows. The thermodynamically most stable and most relevant species for each oxidation state are highlighted with a box. For simplicity, most pathways are depicted as only proceeding in one direction; however, in principle, all redox and structural processes are (reversible) equilibria.

The redox properties of **TX-Acr** were then investigated in detail by cyclic voltammetry (CV). In the anodic scan direction, a single wave, corresponding to the two-electron oxidation to **TX-Acr**^**2+**^, was observed at +0.355 V vs Fc/Fc^+^ in DCM (Figure 5A). Gratifyingly, this oxidation potential lies in between that of its parent congeners **DMBA** (−0.020 V) and **BTX** (+0.915 V), validating the “mix and match” design principle (Figure 5B), as also previously observed for an extended, unsymmetrically substituted anthraquinodimethane-based switch.^57^ Similarly, the reduction of the **TX-Acr**^**2+**^ dication proceeds at intermediate potentials but, in sharp contrast to both of the symmetric switches, proceeds via two clearly separated one-electron reduction waves at potentials of −0.180 and −0.440 V, corresponding to significant redox hysteresis of 535 and 795 mV, respectively (Table 1, Figure 6A). This large separation of oxidation and reduction processes is in good agreement with the behavior of other dynamic redox switches, including **BTX**(39) and lucigenin,^45^ and is indicative of a significant geometric rearrangement associated with the redox events.

**Table 1:** Redox Properties of the BAE Redox Switches Determined by CV in DCM, 100 mM TBAPF_6_ Containing 0.5 mM Neutral Switches (at ν = 0.1 V/s)

	**DMBA**	**TX-Acr**	**BTX**
*E*_pa_ (V)^a^	–0.020	+0.355	+0.915
*E*_pc_ (V)^a^	–0.730^b^	–0.440, −0.180	+0.010
hysteresis (V)	0.710	0.795, 0.535	0.905

a Anodic and cathodic peak potentials vs Fc/Fc^+^.

b Broad peak comprised of two overlapping waves.

Specifically, the interconversion between the neutral, *anti-*folded state and the dicationic, orthogonal state requires at least one passage of the opposite rotor halves in the fjord region, which is, however, sterically impeded and thus associated with a significant energy barrier (Figure 6A).

As a result, the oxidation of **TX-Acr** to **TX-Acr**^**2+**^ proceeds via an ECE (electron transfer, chemical conversion, electron transfer) mechanism:^45,46^ removal of one electron from the neutral *anti-*folded **TX-Acr(Af)** occurs only at comparably high potentials and first, transiently, generates the monoradical cation in the *anti-*folded state **TX-Acr**^**+●**^**(Af)**, which is however thermodynamically much less stable than the twisted (tw) conformation of this species and thus very quickly rearranges to **TX-Acr**^**+●**^**(Tw)** (*k*_2_, Figure 6B). The oxidation potential of this twisted conformer is, however, lower than that of **TX-Acr(Af)** such that the second one-electron oxidation proceeds more readily and, with minimal steric constraints, generates the orthogonal **TX-Acr**^**2+**^**(Ort)** (via the *k*_4_ pathway). Due to this potential inversion, i.e., *E*_Tw,2_ < *E*_Af,1_, the oxidation of the neutral **TX-Acr(Af)** always proceeds via simultaneous two-electron oxidation, and, in this “forward” direction, no radical cation intermediate can be accessed.

**Figure 7:**
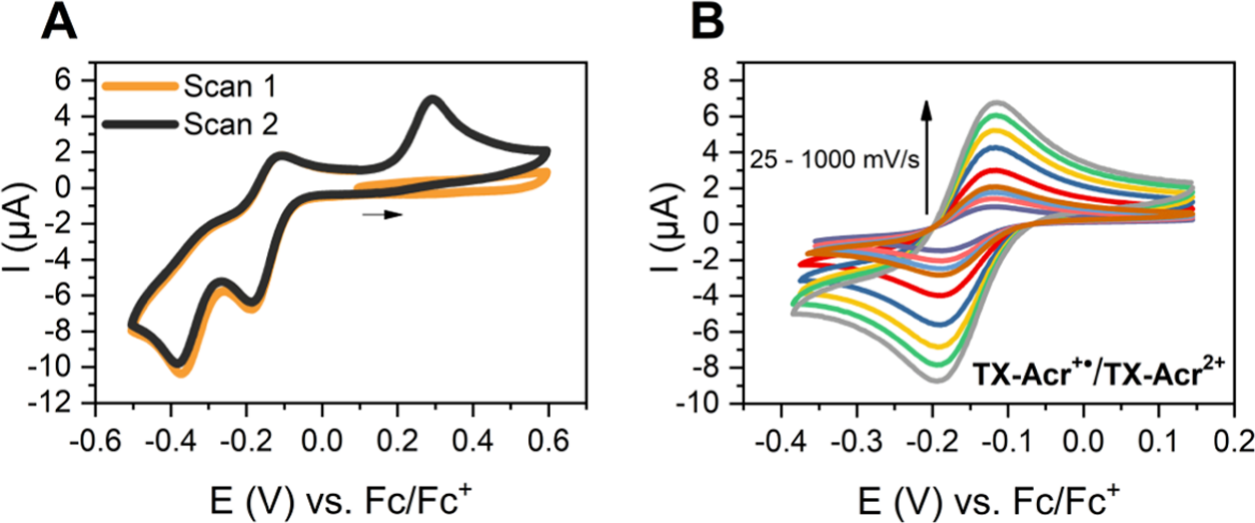
CVs of 0.5 mM **TX-Acr**^**2+**^**(ClO**_**4**_**)**_**2**_ in CH_3_CN, 100 mM TBAPF_6_. (A) At a scan rate of ν = 0.1 V/s over a wide potential range, displaying all redox waves using a GC working electrode. The black arrow indicates the initial direction of the first scan. (B) CVs at varying scan rates of only the reversible **TX-Acr**^**●+**^/**TX-Acr**^**2+**^ couple using a Pt working electrode, where the different redox waves are more separated than with GC. For further information, see Figure S25.

In contrast, one-electron reduction of **TX-Acr**^**2+**^**(Ort)** generates **TX-Acr**^**+●**^**(Tw)** (via *E*_Ort,2_ and *k*_3_), which, within a narrow potential window, is the thermodynamically most stable species. As depicted in Figure 6A, this one-electron reduction is expected to occur on the thioxanthylium rotor, thereby generating the neutral radical species of this switch half, while the acridinium motif remains intact, which was corroborated by spectroelectrochemical studies (vide infra and Figure 9). This radical cation **TX-Acr**^**+●**^**(Tw)** can be either fully reversibly reoxidized to the dication (vide infra, Figure 7) or irreversibly reduced to **TX-Acr(Tw)** (*E*_Tw,1_), which immediately rearranges back to the more stable *anti-*folded **TX-Acr(Af)** (*k*_1_, in an overall EEC mechanism), thereby closing the cycle. This was corroborated by calculations at the r^2^SCAN-3c level of theory, which confirms that the lowest-energy conformer for the radical cation state **TX-Acr**^**+●**^ is indeed a twisted structure (Figure S12), while the *anti*- and *syn*-folded conformers of this redox state are significantly less stable (by 49 and 97 kJ/mol, respectively).

Further support for these mechanisms was obtained by voltammetric studies at ∼−75 °C, where the conformational rearrangement from **TX-Acr**^**+●**^**(Af)** to **TX-Acr**^**+●**^**(Tw)** (i.e., *k*_2_) is sufficiently slowed down (Figure S21). In this case, the CV for oxidation of **TX-Acr** displays an additional redox wave at more positive potentials, corresponding to further one-electron oxidation of **TX-Acr**^**+●**^**(Af)** to the corresponding dication in the *anti-*folded state (**TX-Acr**^**2+**^**(Af)**, *E*_Af,2_ pathway in Figure 6B).

This redox switch thus displays not only a pronounced hysteresis but also pathway dependence. This is also manifested by a complete lack of redox activity of **TX-Acr** in the potential window cathodic of its oxidation peak if no **TX-Acr**^**2+**^ was prior generated (see, for example, the first CV scan in Figure 5A and vice versa in Figure 7A). Furthermore, the intermediate radical cation state can only be accessed by reduction of the dication but not by oxidation of the neutral state (Figure 6A). Importantly, this unique behavior cannot be observed in classic, reversible redox couples and can be exploited in molecular information storage and processing systems,^34,58−61^ including neuromorphic computing, where hysteresis is a crucial prerequisite.^62^

Starting from **TX-Acr**^**2+**^, CV experiments in CH_3_CN reveal, as expected, a qualitatively similar behavior as discussed above: pronounced hysteresis, two one-electron reductions, and a two-electron oxidation at +0.29 V that is only observed if **TX-Acr** was generated beforehand (Figure 7A). Under these conditions, it is also possible to study the intermediate radical cation state in more detail.^63^ As shown in Figure 7B, the first reduction of **TX-Acr**^**2+**^ to **TX-Acr**^**+●**^ is not only sufficiently separated from the second one-electron reduction but is also the only fully electrochemically reversible and bidirectional process in the overall redox switching cycle.

While all other redox processes proceed with significant geometric rearrangements that lead to “ratcheting” and irreversibility, the barrierless interconversion between the orthogonal dication and the twisted monoradical cation endows the **TX-Acr**^**+●**^/**TX-Acr**^**2+**^ redox couple with a very high degree of reversibility. This is evidenced by a near unity ratio of peak currents and a low peak separation of 70 mV (at ν = 25 mV/s), indicating a high chemical stability of **TX-Acr**^**+●**^. In addition, the peak currents depend linearly on the square root of the scan rate, indicating a diffusion-controlled process, as is indeed also the case for the redox processes of **TX-Acr** in DCM (see Figures S22–S24).

In this context, it is particularly noteworthy that the intermediate radical cation state can only be quantitatively generated and spectroscopically studied (vide infra) for the mixed **TX-Acr** switch; for **BTX/BTX**^**2+**^, both oxidation and reduction proceed via a two-electron mechanism without detectable radical intermediates,^39,40^ while for **DMBA/Luc**^**2+**^, the reduction also proceeds via an EEC mechanism, however with almost identical reduction potentials for the **Luc**^**2+**^ and **Luc**^**+●**^ states.^45^ This is also reflected in the broader reduction wave for **Luc**^**2+**^ (Figure 3B), which is composed of two overlapping waves. As a result, the intermediate **Luc**^**+●**^ can only be generated in small quantities at the onset of the reduction wave and is additionally prone to disproportionation.^45^ Alternatively, DYREX switches with more than two redox states (and colors) can also be prepared by a combination of multiple DYREX units, as demonstrated by Ishigaki, Suzuki, and co-workers.^64,65^ Of additional note is that the redox switching behavior of **TX-Acr**, comprising an ECE mechanism in one direction and an EEC mechanism in the reverse direction, is analogous to that of many DYREX systems based on reversible C–C single bond formation/breakage.^34^

### Optical Properties

**Figure 8:**
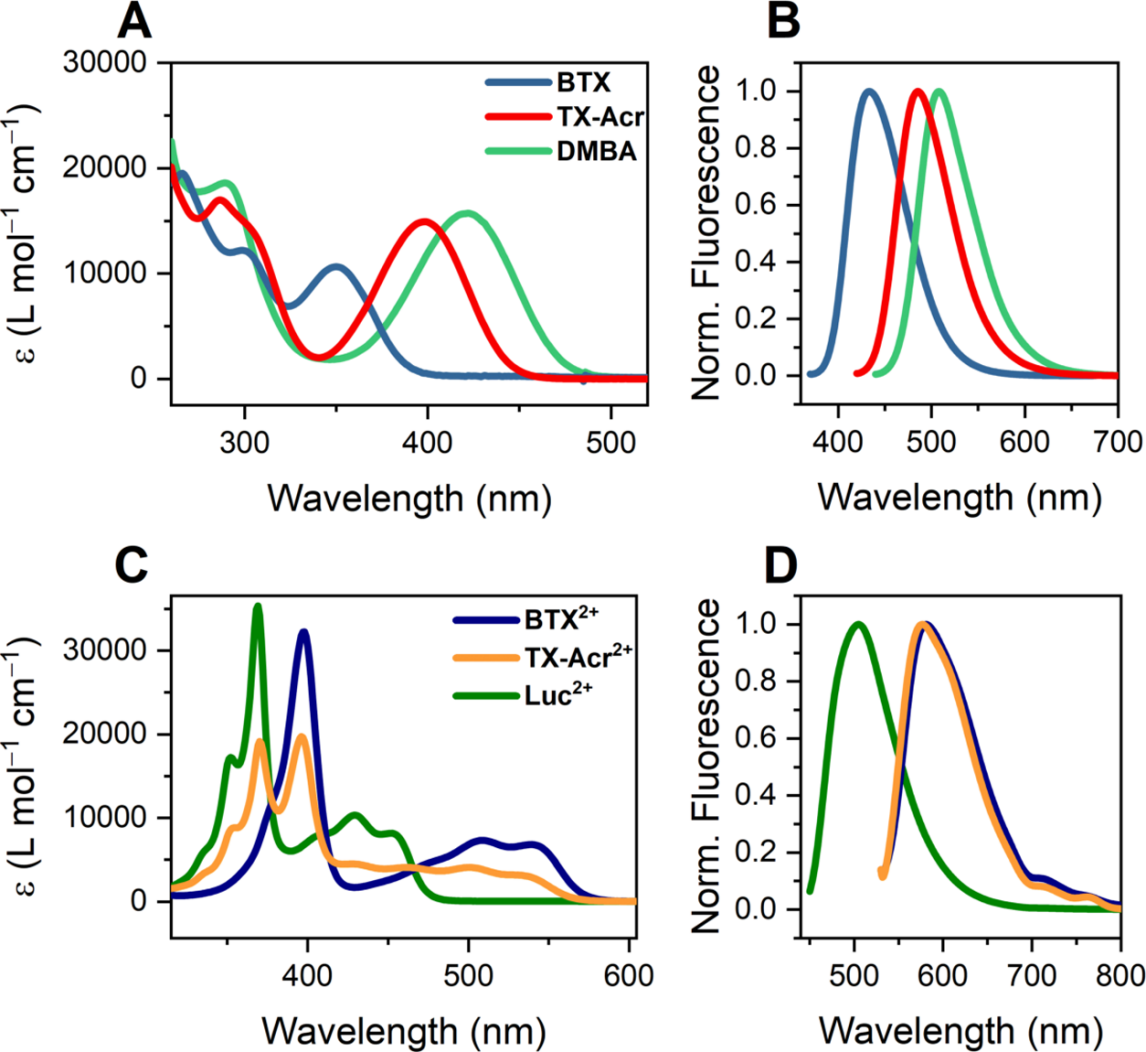
UV–vis (A, C) and normalized fluorescence emission spectra (B, D) of the neutral switches in DCM (A, B) and the dicationic switches (as perchlorate salts) in CH_3_CN (C, D).

Mirroring the voltammetric behavior, the optical properties of the neutral **TX-Acr** also lie between those of its symmetric parent analogs. Specifically, the absorption maximum of the lowest energy band of **TX-Acr** lies at λ_Max,Abs_ = 398 nm, while **BTX** (350 nm) and **DMBA** (422 nm) are more blue- and red-shifted, respectively (Figure 8A). The same trend is also observed for their emission spectra, where **TX-Acr** shows an intermediate fluorescence emission maximum at λ_Max,Em_ = 485 nm (Figure 8B and Table 2), and all compounds display almost identical Stokes shifts of ∼85 nm. These observations are in good agreement with the chromophore spanning the whole molecule, i.e., both halves are conjugated through the central double bond and contribute to the overall optical properties. In the neutral state, **TX-Acr** thus possesses optical properties that are the average of its parent motifs.

**Table 2:** Photophysical Properties of Neutral and Dicationic Switches in DCM and CH_3_CN, Respectively

	**DMBA**	**TX-Acr**	**BTX**	**Luc**^**2+**^	**TX-Acr**^**2+**^	**BTX**^**2+**^
λ_Max,Abs_ (nm)^a^	422	398	350	429	500	509
ε (M^–1^ cm^–1^)^a^	15,760	14,940	10,670	10,330	4080	7310
λ_Max,Em_ (nm)	508	485	433	505	577	581

a Lowest energy band. For the dications, this band is broader and is split. Here, only the main peak is reported.

Notably, this behavior is not observed for the respective dications. As shown in Figure 8C, the absorbance bands of **TX-Acr**^**2+**^ do not lie between those of **BTX**^**2+**^ and **Luc**^**2+**^ but are rather a superimposition of these symmetric compounds. This can be rationalized when considering that in the orthogonal dication state, the two switch halves are electronically isolated and not conjugated, such that **TX-Acr**^**2+**^ displays the characteristics of both the acridinium and thioxanthylium rotors. This is also reflected in its fluorescence emission spectra, whereby λ_Max,Em_ = 577 nm is very close to that of **BTX**^**2+**^ (581 nm), indicative of fluorescence emission from only the thioxanthylium rotor.

Of additional note in this context is that only **TX-Acr**^**(2+)**^ and **BTX**^**(2+)**^ display a significant difference in fluorescence emission between their neutral and dicationic states, with red shifts of 92 and 148 nm, respectively. In contrast, **DMBA** and **Luc**^**2+**^ display virtually identical emission profiles and thus cannot be used as a redox-response fluorescence switch. Additionally, only for the mixed switching system is an additional third (nonfluorescent) state quantitatively accessible via redox stimuli (**TX-Acr**^**+●**^, vide infra), allowing modulation of emission between three different states. **BTX** can, in principle, also switch between three different fluorescent states, including a nonfluorescent, neutral *syn*-folded state. However, while this state can be transiently generated electrochemically, it quickly thermally relaxes back to the blue-fluorescent *anti*-folded state. In contrast, **TX-Acr** displays three fully stable redox-interconvertible states with distinct fluorescence.

### Spectroelectrochemistry

**Figure 9:**
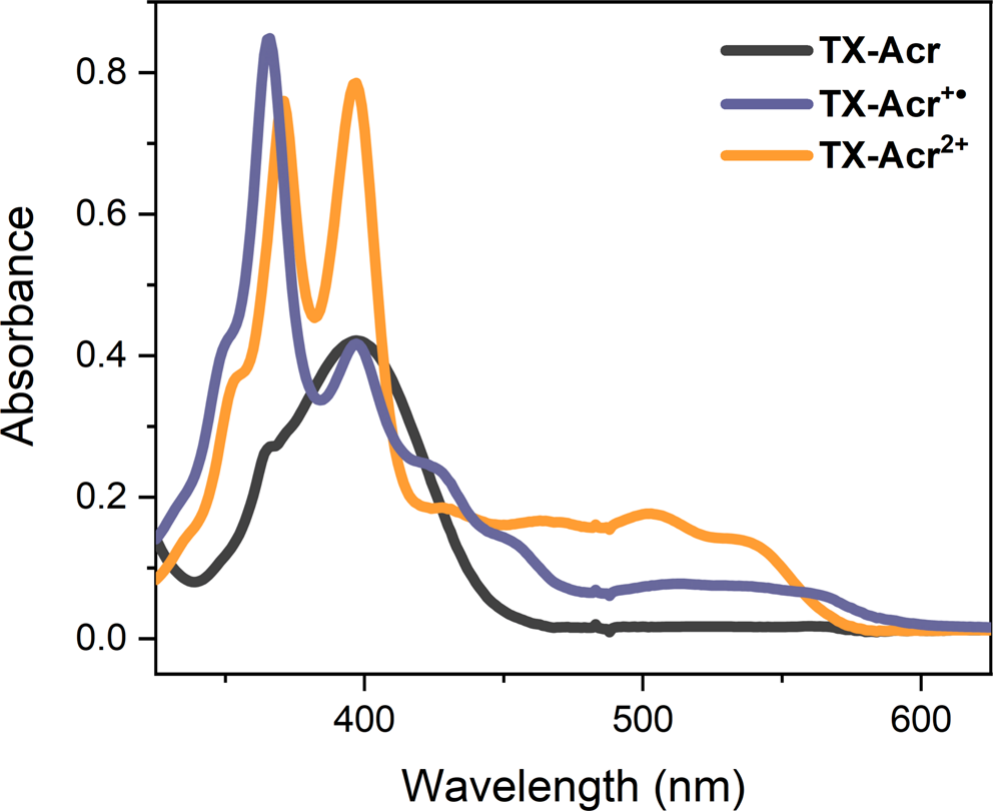
UV–vis spectra of all three redox states of **TX-Acr** in DCM/CH_3_CN 1:1, 200 mM TBAPF_6_ generated spectroelectrochemically.

To further study this third monoradical cation switching state, spectroelectrochemical experiments were carried out. As shown in Figure 9, the UV–vis spectrum of the electrochemically generated **TX-Acr**^**+●**^ displays absorbance bands that are qualitatively similar to that of the dication. Specifically, a broad, low energy absorbance extends up to ∼590 nm, corresponding to a small bathochromic shift in comparison to that of the dication (up to ∼570 nm). Similarly, a strong absorbance is observed at 366 nm, weakly hypsochromically shifted from an initial value of 371 nm. In contrast, the initially strong band of **TX-Acr**^**2+**^ at 397 nm is much weaker in the radical cation. As shown in Figure 8C, this band corresponds to the thioxanthylium rotor; the disappearance of this band upon reduction to **TX-Acr**^**+●**^ strongly suggests that the radical is mainly located on the thioxanthene rotor such that the cationic acridinium rotor remains “intact” (as depicted in Figure 6A). Indeed, a direct comparison of the normalized absorbance spectra of the monoradical cation with **BTX**^**2+**^ and **Luc**^**2+**^ (Figure S26) further confirms that the absorbance of **TX-Acr**^**+●**^ most closely resembles that of **Luc**^**2+**^. These observations are also in good agreement with the higher oxidation potential of the thioxanthene rotor.

**Figure 10:**
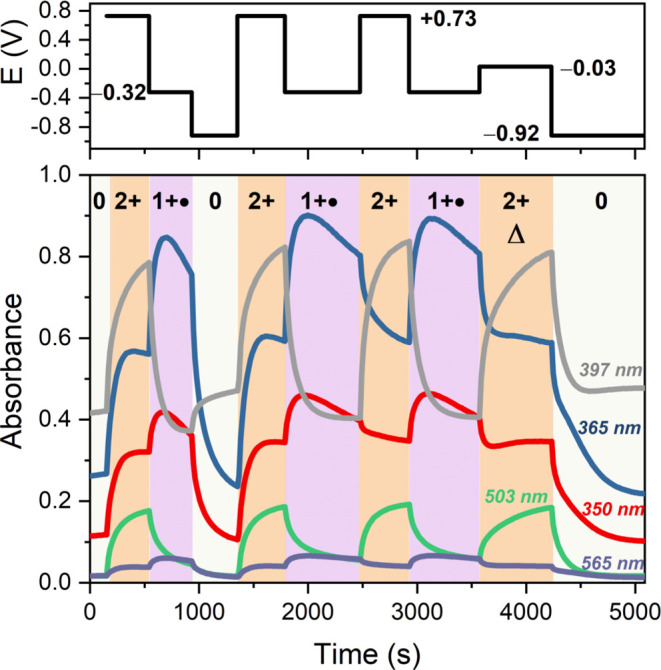
Changes in absorbance of **TX-Acr** in DCM/CH_3_CN 1:1, 200 mM TBAPF_6_ during spectroelectrochemical cycling using a Pt mesh working electrode between all three different redox states at different potentials (vs Fc/Fc^+^) as indicated at the top. The cycle marked with “Δ” was carried out by oxidation of the radical cation at a potential that is less anodic than the initial oxidation potential of **TX-Acr** (see Figures 7 and S25).

In addition, these spectroelectrochemical studies confirmed a very high degree of reversibility for the redox switching of **TX-Acr** across all three oxidation states; see Figures 10 and S27. Of particular note is, as briefly discussed above, that the switch displays an important pathway dependence; the monoradical cation state can only be accessed via reduction of the dication and not via oxidation of the neutral *anti-*folded state, as also summarized in Figure 6A. Oxidation of this latter state always proceeds via simultaneous two-electron removal with no appreciable generation of any radical cation intermediate. This was also confirmed by clean isosbestic points in the absorbance spectra upon oxidation of **TX-Acr** (Figure S28). Similarly, conversion from **TX-Acr**^**+●**^ to either **TX-Acr** or **TX-Acr**^**2+**^ proceeds via clean isosbestic points (Figures S29 and S30). Only the one-electron reduction from **TX-Acr**^**2+**^ to **TX-Acr**^**+●**^ displays somewhat more complex behavior that is characterized by an initial, fast change in absorbance resulting in the purple spectrum in Figure 9, followed by a less pronounced further change as shown in Figure S31. This slower secondary process can be ascribed to a partial further reduction to neutral **TX-Acr**.

In addition to the pathway dependence, it should also be noted that the (re)generation of the dicationic switching state can be carried out under different conditions: the “initial” two-electron oxidation of the neutral *anti-*folded **TX-Acr** can, due to significant hysteresis, proceed only at potentials close to, or above, ∼+0.35 V (Figure 3). In contrast, oxidation of **TX-Acr**^**+●**^ can proceed at much milder potentials (∼−0.1 V, Figure 5), as shown in the penultimate spectroelectrochemical cycle (“Δ”, Figure 10).

To study the emission properties of **TX-Acr**^**+●**^, fluorescence-spectroelectrochemical studies were carried out. As shown in Figure S32, oxidation of **TX-Acr** to **TX-Acr**^**2+**^ induced the expected switching from strong green to weak orange fluorescence emission, in good agreement with studies on the isolated compounds (Figure 8B,D). Upon reduction to **TX-Acr**^**+●**^, the orange emission peak from the dication vanished, indicating that this state is nonemissive, as expected for a radical. As a result, **TX-Acr** represents, in contrast to **BTX** or **DMBA**, a three-state fluorescent redox switch. Such multistate switching, especially that of different parameters (here: optical, geometric, redox state) remains rare but is highly sought after in various applications, such as advanced molecular logic gates.^66−69^

## Conclusions

The development of multistate small-molecule DYREX switches that possess a high degree of redox stability, distinct (conformational) switching states, and favorable redox potentials is of fundamental importance but remains a significant challenge. Herein, we demonstrated that the mixed **TX-Acr**, containing both a thioxanthone and an acridone-derived rotor half, is an easily synthetically accessible, highly reversible, and versatile conformational redox switch. Its neutral *anti-*folded and dicationic orthogonal states can be isolated as stable solids and interconverted at mild potentials with high fidelity. This is expected to enable a much larger range of applications than is currently possible with its symmetric parent congeners, **BTX** and **DMBA**, both of which possess less favorable redox potentials. In addition, only for **TX-Acr** is a third stable redox state, the twisted monoradical cation, quantitatively accessible. As a result, **TX-Acr** is a robust three-state redox switch with different absorbance, fluorescence, polarities, and geometries between all states. Due to the significant conformational rearrangements between the different switching states, this system also displays a significant redox hysteresis and notable pathway dependence; conformational ratcheting following electron transfers effectively endows sequence directionality to most interconversions.

These findings pave the way for the exploration of novel DYREX switches whose switching properties can be judiciously tuned by the mix and match combination of various DYREX halves. Consequently, these systems may open new opportunities for applications in multiredox or energy storage systems,^41,70^ magnetic/radical switching,^38,71^ neuromorphic computing,^62^ sensing,^72^ photoredox catalyses,^73^ as well as molecular machines and actuators.

## Supporting Information Available

Further details on experimental procedures, NMR spectra, (spectro)electrochemical data, computational data, and structural analysis (PDF)
XYZ_coordinates_conformational_analysis (XYZ)
XYZ_coordinates_syn-anti_relaxation (XYZ)

## Supplementary Material

ja4c08284_si_001.pdf

ja4c08284_si_002.xyz

ja4c08284_si_003.xyz

## Footnotes

a We herein define DYREX as any system that undergoes significant geometric rearrangements and/or reversible bond cleavage upon redox, regardless of the specific mechanism. For further discussions see refs (34,36,74).
