# Supplementary material for: Mix and Match Tuning of the Conformational and Multistate Redox Switching Properties of an Overcrowded Alkene
Source: J Am Chem Soc. 2024 Sep 13;146(38):26275–85. doi: 10.1021/jacs.4c08284 (PMC11440491; doi:10.1021/jacs.4c08284)
Supplement: Supplementary file 1 — ja4c08284_si_001.pdf [file ja4c08284_si_001.pdf]

Supporting Information

Mix and Match Tuning of the Conformational and Multistate Redox Switching Properties of an Overcrowded Alkene

Robert Hein, Charlotte N. Stindt and Ben L. Feringa**

Stratingh Institute for Chemistry, University of Groningen, Nijenborgh 4, 9747AG Groningen, The Netherlands
*r.hein@rug.nl, *b.l.feringa@rug.nl

Contents

1. Experimental 2
2. Synthesis 3
3. X-ray structural data 11
4. Computational analysis 22
5. ^{1}H NMR Irradiation Studies 26
6. Voltammetric Characterisation 30
7. Spectroelectrochemistry 32
8. References 36

1. Experimental

General

All chemicals and solvents were obtained from commercial suppliers and used as received. $TBAPF_6$ was of electrochemical grade and obtained from Sigma Aldrich. Lucigenin was obtained as a nitrate salt from TCI chemicals. Unless otherwise noted, HPLC grade solvents were used.

All experiments were carried out at room temperature under ambient conditions, unless otherwise stated. NMR experiments were conducted using a Varian Mercury Plus (400 MHz), an Agilent MR 400 (400 MHz), a Varian Inova 500 (500 MHz) or a Bruker Avance Neo 600 (600 MHz) spectrometer and spectra were referenced to the residual solvent signal. High-resolution mass spectra were recorded on a Thermofisher LTQ Orbitrap XL.

Electrochemical Measurements

All electrochemical experiments were carried out using a three-electrode setup using a Palmsense 4 potentiostat. Pt wire was used as a counter electrode throughout. Unless otherwise stated a glassy carbon disk working electrode (3 mm diameter) was used. In select cases a Pt disk working electrode (1.6 mm diameter) was used as indicated. Prior to each experiment the working electrode surface was polished using a 0.05 µm alumina slurry. In organic solvents a non-aqueous $Ag/AgNO_3$ reference electrode (10 mM $AgNO_3$ in CH_3CN, 100 mM $TBAPF_6$) was employed. For low-temperature experiments a Ag wire pseudo-reference electrode was typically used and the solution was cooled using a dry ice/acetone bath. The solution was kept under an Argon blanket to avoid water condensation. Unless otherwise stated, a scan rate of 100 mV/s was used for all CV experiments, and experiments were carried out under ambient conditions in HPLC grade solvents and in the presence of oxygen. $TBAPF_6$ was used as supporting electrolyte in all cases (100 mM for standard voltametric experiments and 200 mM for spectroelectrochemistry). No iR compensation was carried out. The uncompensated solution resistance in DCM, 100 mM $TBAPF_6$ was ~1 kΩ as determined by electrochemical impedance spectroscopy at high frequencies (~100 kHz, 10 mV amplitude, E_{dc} = OCP).

Optical Measurements

UV-Vis spectra were recorded on an Agilent Cary 8454 spectrophotometer using 1 x 1 cm quartz cuvettes, or for spectroelectrochemical experiments in a quartz spectro-electrochemical cell with 1 mm pathlength (ALS Japan). Fluorescence spectra were obtained on a JASCO FP-6200. Fluorescence spectra were recorded using 5 nm excitation and 10 nm emission slit widths. All samples were measured at a concentration of 2.5 µM. The following

excitation wavelengths were used for standard characterization (Figures 8B and 8D): 425 nm, 510 nm, 510 nm for Luc^{2+}, TX-Acr^{2+} and BTX^{2+}, respectively and 350 nm, 400 nm, 420 nm, for BTX, TX-Acr and DMBA, respectively.

Spectroelectrochemistry

All spectroelectrochemical experiments were carried out using a Pt mesh electrode, non-aqueous Ag/$AgNO_3$ reference electrode (10 mM $AgNO_3$ in CH_3CN, 100 mM $TBAPF_6$) and a Pt wire counter electrode. A 1 mm pathlength spectroelectrochemical cuvette (ALS Japan) was used in all cases. For UV-Vis spectroelectrochemical experiments a 320 nm cutoff filter was also used. To ensure complete electrolysis, potentials that were at least 150 mV anodic or cathodic of the respective redox waves were applied, typically for 10-15 min per cycle. Spectra were recorded every 15 s (UV-Vis) or 60 s (fluorescence). For fluorescence spectroelectrochemical measurements an excitation wavelength of 400 nm was used.

^{1}H NMR Irradiation Studies

NMR irradiation experiments were carried out using a Varian Inova 500 spectrometer. The sample was cooled to ~–90 °C and irradiated *in situ* with a Thorlabs LED (M395) with a 1500 µm optical fiber (FT1500UMT) to guide the light directly into the NMR tube.

2. Synthesis

BTX[1] and 10-methyl-9,10-dihydroacridine[2] were prepared according to literature procedures.

TX-Acr

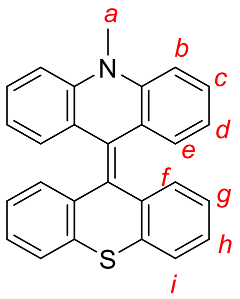

10-Methyl-9,10-dihydroacridine (195 mg, 1 mmol, 1 equiv.) was dissolved in 10 mL dry THF in a Schlenk flask and cooled to –70 °C. To this solution 0.45 mL *n*Buli (2.5 M in hexanes, 1.12 mmol, 1.12 equiv.) was added and the mixture allowed to react while cooled in an ice bath for 30 min. Afterwards the solution was cooled to –60 °C and 0.14 mL TMSCl added (1.11 mmol,

1.11 equiv.) and the reaction allowed to proceed for 15 min. Another 0.48 mL of *n*Buli (2.5 M in hexanes, 1.2 mmol, 1.2 equiv.) was added at –60 °C and the mixture stirred for 1 h at room temperature. Next, the solution was cooled to –50 °C and 212 mg thioxanthen-9-one was added. The mixture was allowed to react at room temperature overnight and quenched by pouring into 50 mL saturated aqueous NH_4Cl. The crude product was then extracted with DCM (3 x 50 mL) and the combined organic phases washed with water and then brine. The product was then purified by column chromatography on silica (pentane/DCM using a gradient from 0 to 100% DCM). This afforded 134 mg **TX-Acr** (34%) as a bright yellow solid.

^{1}H NMR (600 MHz, $CDCl_3$) δ 7.59 – 7.52 (m, 2H), 7.20 (ddd, *J* = 8.5, 7.1, 1.5 Hz, 2H), 7.12 (ddd, *J* = 7.8, 6.8, 1.8 Hz, 2H), 7.06 (dd, *J* = 8.2, 1.1 Hz, 2H), 7.01 – 6.93 (m, 4H), 6.82 (dd, *J* = 7.8, 1.5 Hz, 2H), 6.68 (ddd, *J* = 7.9, 7.3, 1.1 Hz, 2H), 3.57 (s, 3H).

^{13}C NMR (151 MHz, $CDCl_3$) δ 144.3, 137.6, 136.8, 129.5, 129.2, 129.2, 128.5, 127.8, 127.7, 126.3, 125.9, 123.5, 120.1, 112.4, 33.6.

HR-MS (ESI+): Calculated for $C_{27}H_{19}NS$ $[M]^+$: 389.12327. Found: 389.12288

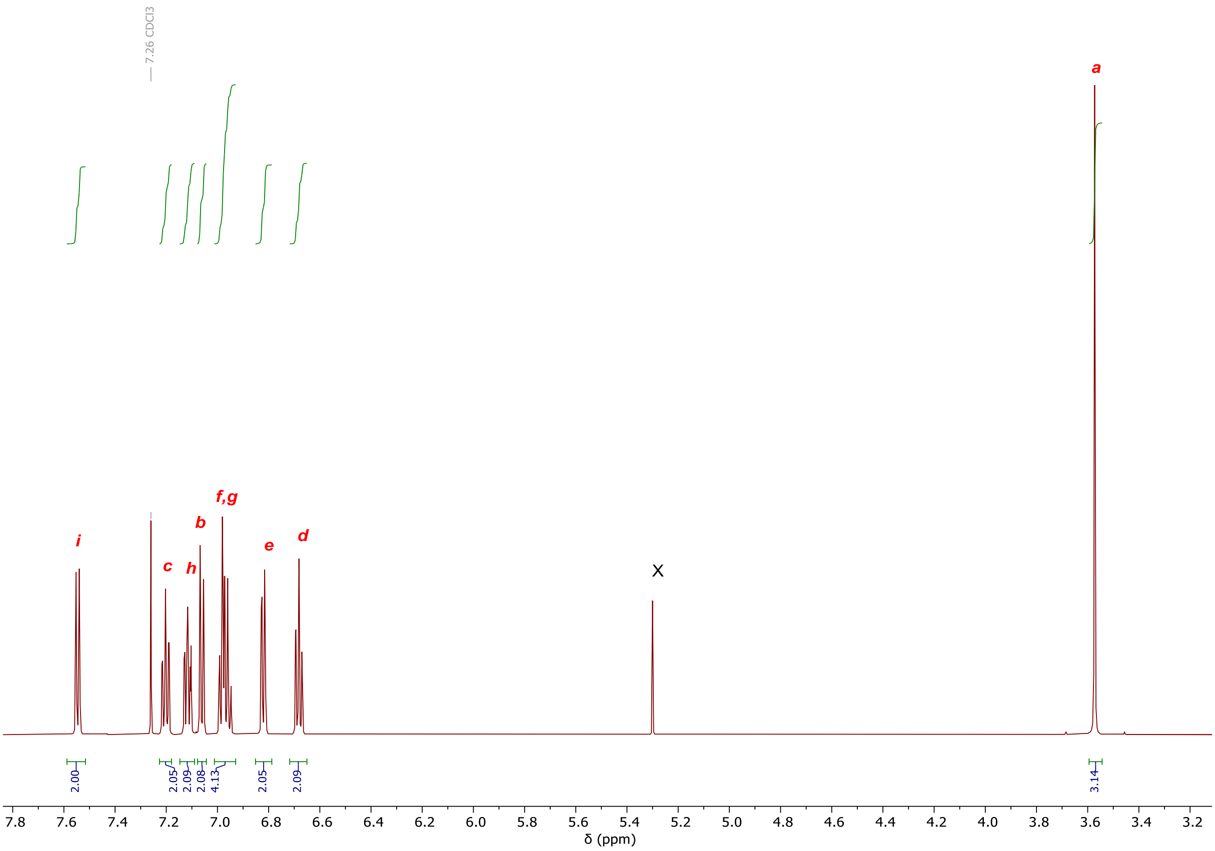

Figure S1. ^{1}H NMR spectrum (600 MHz, $CDCl_3$) of **TX-Acr.** The peak marked X arises from residual DCM.

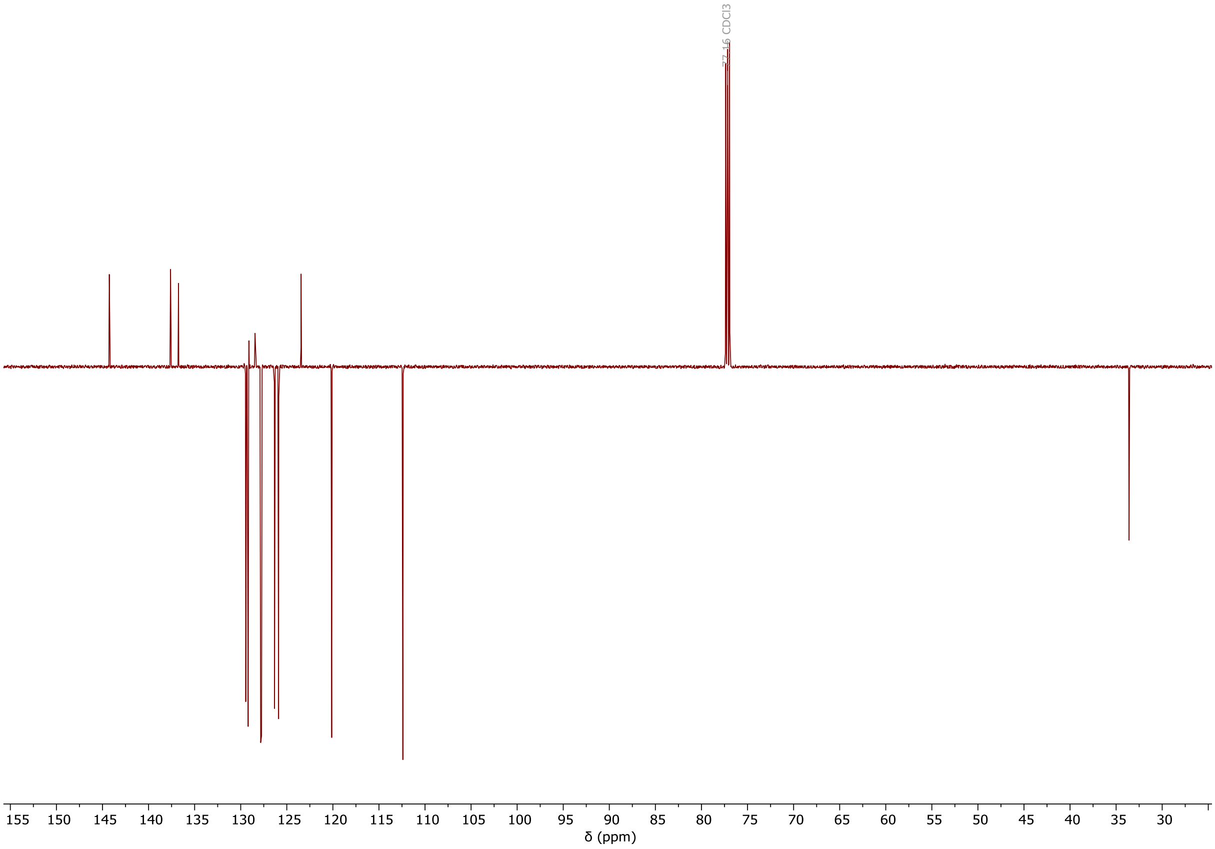

Figure S2. ^{13}C NMR (APT, 151 MHz, $CDCl_3$) of **TX-Acr.**

TX-Acr^{2+}(ClO_4^-)$_2$

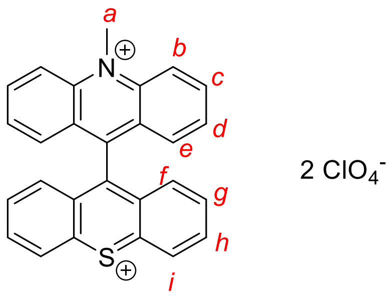

TX-Acr (10 mg, 0.026 mmol, 1 equiv.) was dissolved in 4 mL DCM and 2 mL CH_3CN. To this was added 55 mg $Fe(ClO_4)_3$ (0.154 mmol, 6 equiv.), whereupon the solution turned red immediately. After brief sonication and stirring for 10 min, 3 mL DCM where added and the solution left without stirring for 30 min. The formed red precipitate was collected by filtration over a sintered funnel and washed with DCM. The product was then dissolved off the funnel in CH_3CN and the solvent removed in vacuo. This afforded 13 mg of **TX-Acr^{2+}(ClO_4^-)$_2$** (0.022 mmol, 86%) as a red solid.

^1H NMR (600 MHz, CD_3CN) δ 9.03 (dt, *J* = 8.6, 0.9 Hz, 2H), 8.89 – 8.77 (m, 2H), 8.53 – 8.36 (m, 4H), 7.89 (ddd, *J* = 8.9, 6.8, 1.1 Hz, 2H), 7.76 (dt, *J* = 8.8, 0.9 Hz, 2H), 7.71 (ddd, *J* = 8.7, 6.7, 0.8 Hz, 2H), 7.49 (dd, *J* = 8.8, 1.4 Hz, 2H), 5.06 (s, 3H).

^{13}C NMR (151 MHz, CD_3CN) δ 161.2, 152.5, 150.8, 143.0, 140.5, 139.4, 134.5, 133.6, 130.86, 130.5, 129.6, 129.4, 127.4, 120.5, 40.6.

HR-MS (ESI+): Calculated for $C_{27}H_{19}NS$ $[M]^+$: 389.12327. Found: 389.12309

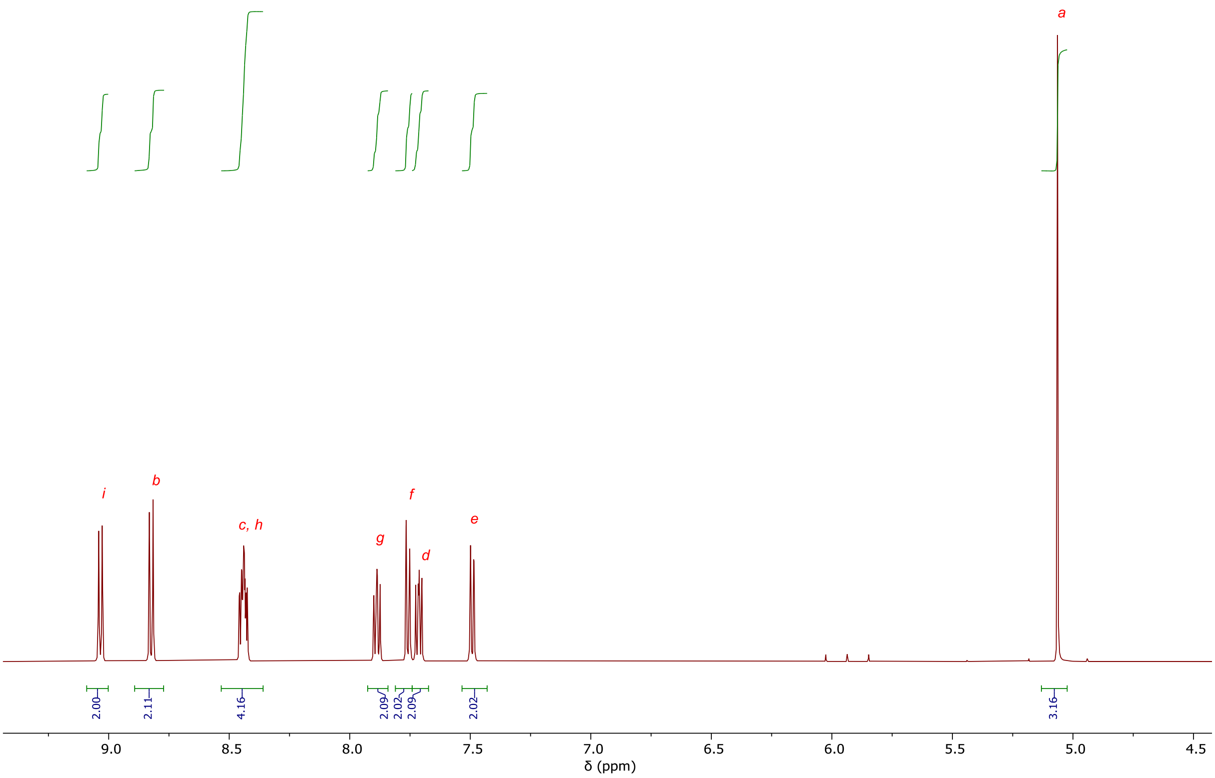

Figure S3. ^1H NMR (600 MHz, CD_3CN) of **TX-Acr^{2+}(ClO_4^-)$_2$**.

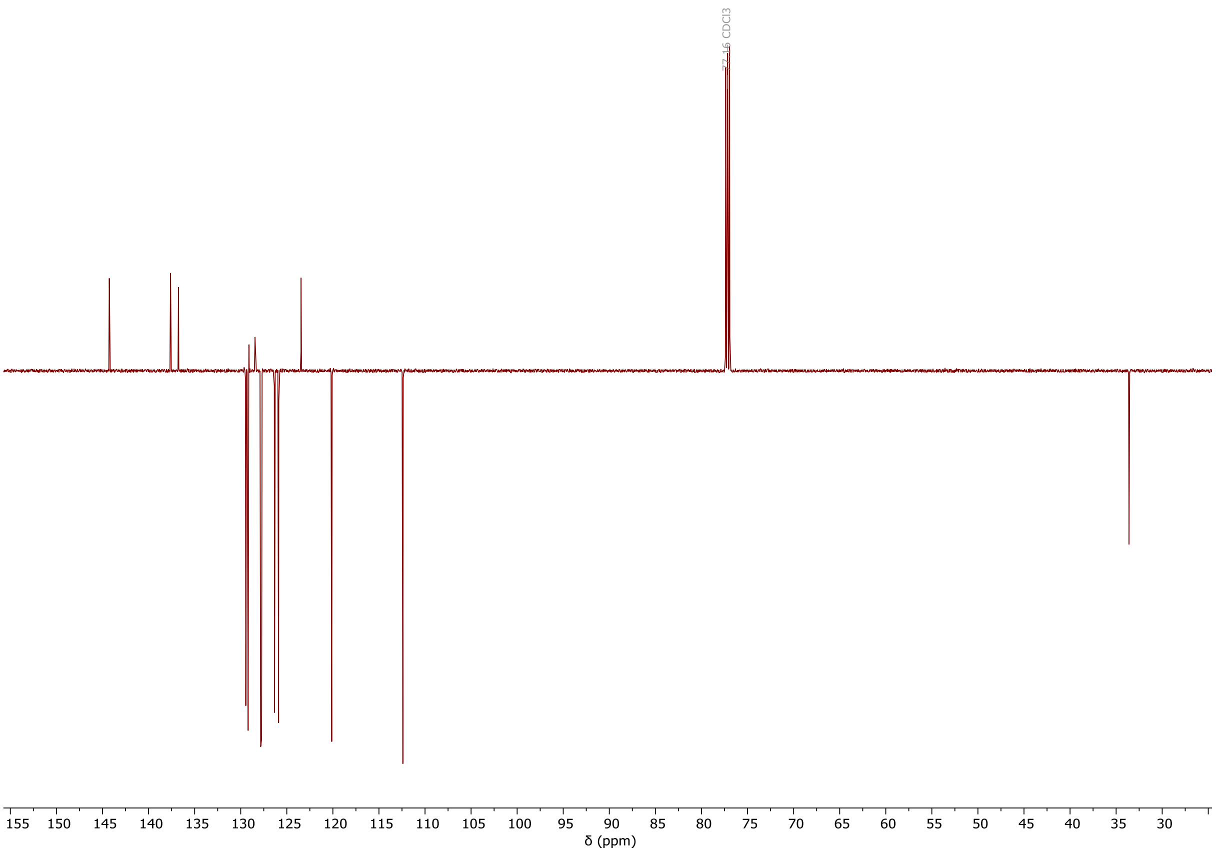

Figure S4. ^{13}C NMR (APT, 151 MHz, CD_3CN) of **TX-Acr^{2+}(ClO_4^-)$_2$**.

BTX^{2+}(ClO_4^-)$_2$

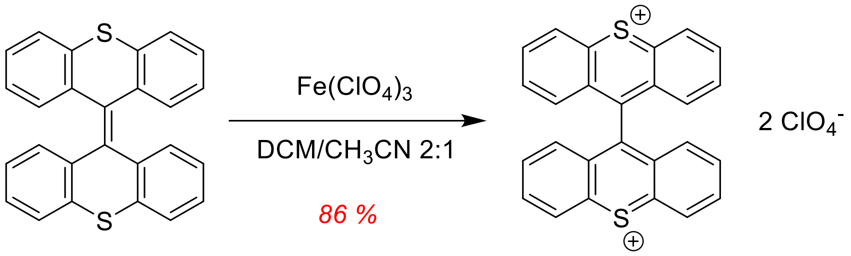

21.5 mg **BTX** (0.055 mmol, 1 equiv.) was suspended in 45 mL DCM/CH_3CN (2:1). To this was added 116 mg $Fe(ClO_4)_3$ (0.329 mmol, 6 equiv.) and the mixture was sonicated for 30 min. The resulting red suspension was left to settle for 30 min and then filtered through a fritted funnel. The red product was then washed with 40 mL DCM/CH_3CN (3:1). The product was dissolved off the frit with CH_3CN and dried under vacuum. This afforded 28 mg (86% yield) of **BTX^{2+}(ClO_4^-)$_2$** as a dark red solid.

^{1}H NMR (400 MHz, CD_3CN) δ 9.06 (d, *J* = 8.6 Hz, 4H), 8.46 (t, 4H), 7.89 (t, *J* = 7.9 Hz, 4H), 7.77 (d, *J* = 8.9 Hz, 4H).

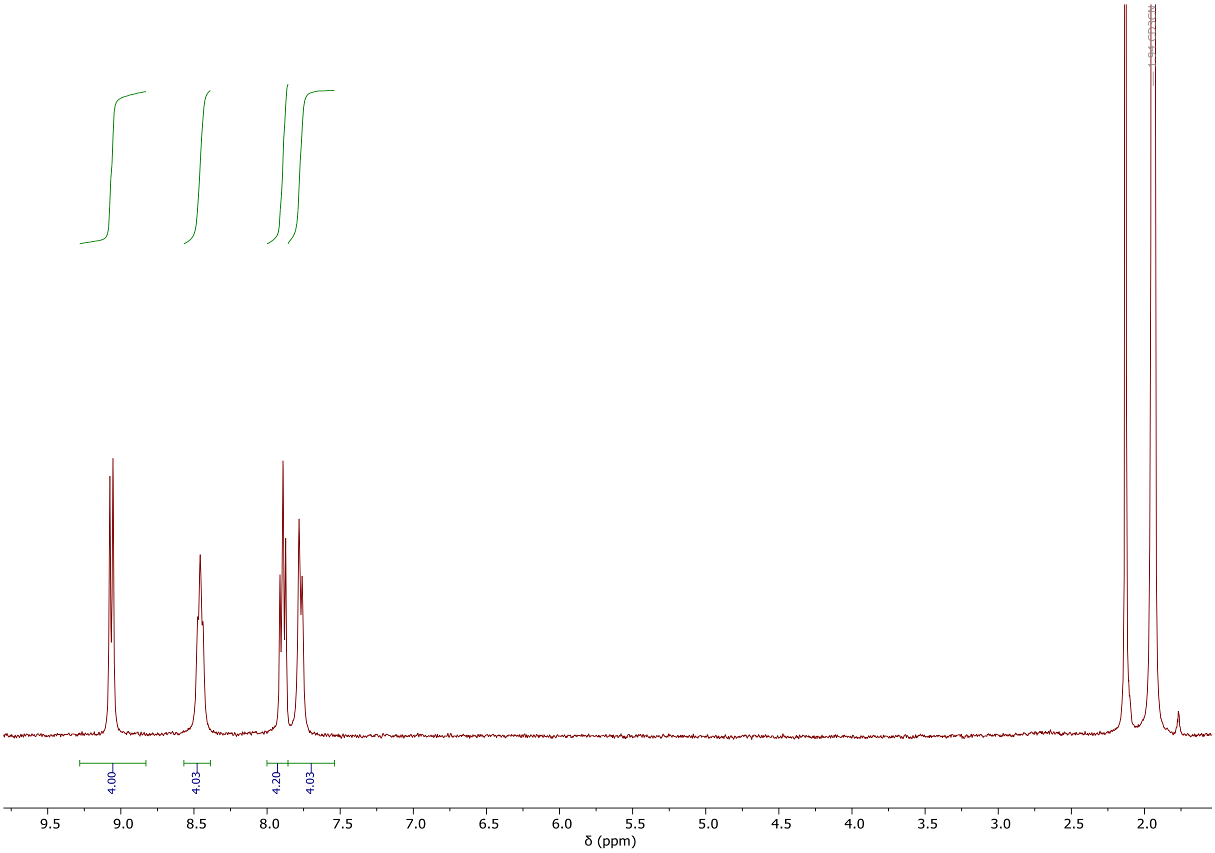

Figure S5. ^{1}H NMR (400 MHz, CD_3CN) of **$BTX^{2+}(ClO_4^-)_2$**.

$Luc^{2+}(ClO_4^-)_2$

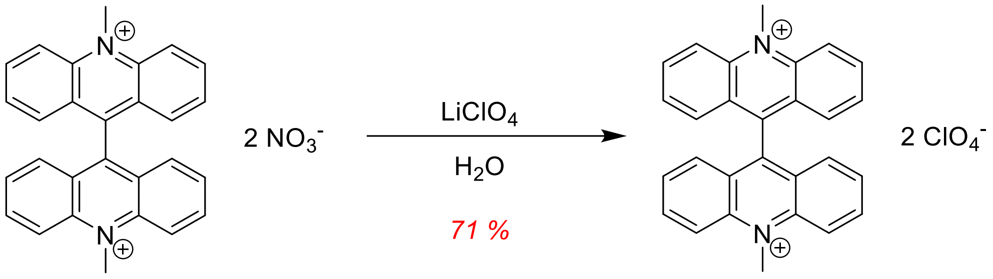

94 mg Lucigenin (as nitrate salt, 0.184 mmol, 1 equiv.) was dissolved in 75 mL H_2O. To this a saturated aqueous solution of $LiClO_4$ was added dropwise until no more precipitation was observed. The precipitate was collected on a fritted funnel and washed with water and then pentane. After drying under vacuum overnight, 76 mg (71% yield) of **$Luc^{2+}(ClO_4^-)_2$** were obtained as a dark yellow solid.

^1H NMR (400 MHz, CD_3CN) δ 8.81 (d, *J* = 9.3 Hz, 4H), 8.44 (s, 4H, broad), 7.73 (t, *J* = 7.7 Hz, 4H), 7.46 (s, 4H, broad), 5.05 (s, 6H, broad).

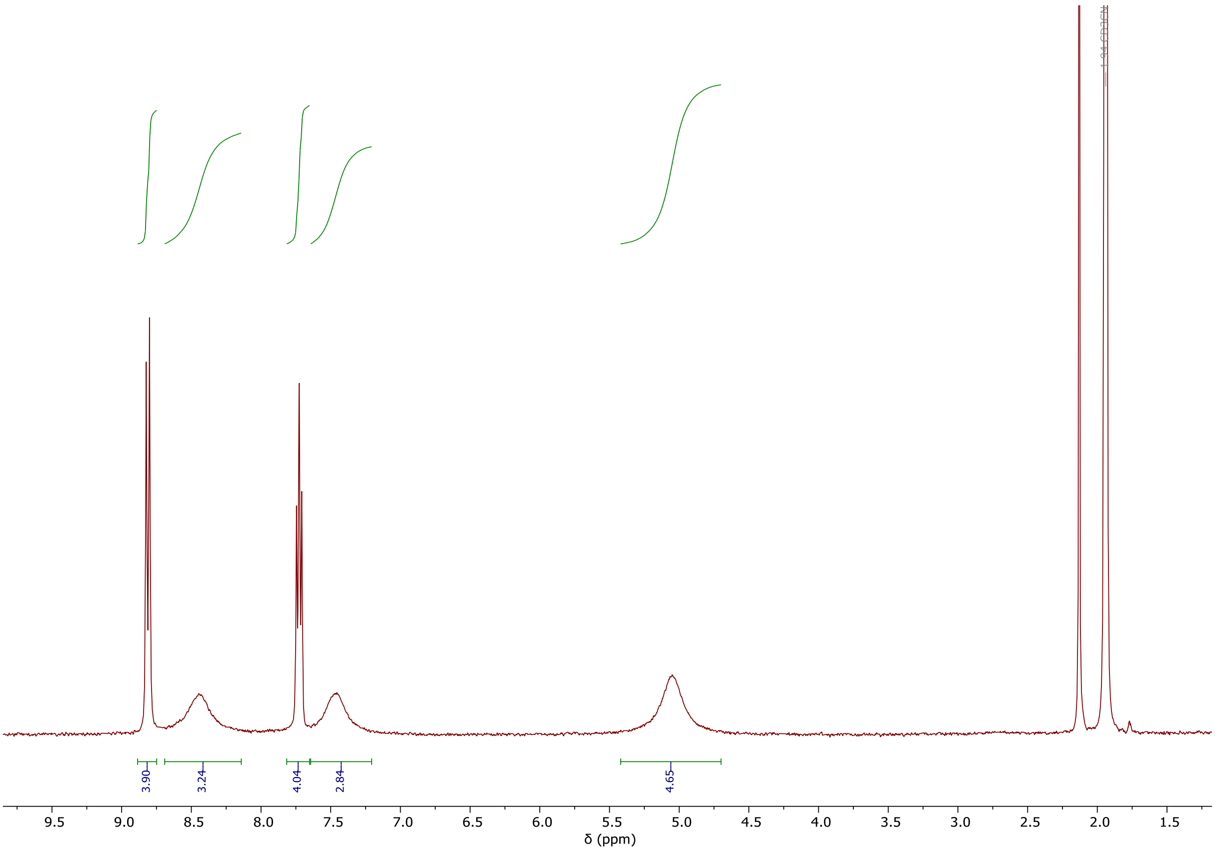

Figure S6. ^1H NMR (400 MHz, CD_3CN) of **$Luc^{2+}(ClO_4^-)_2$**.

DMBA

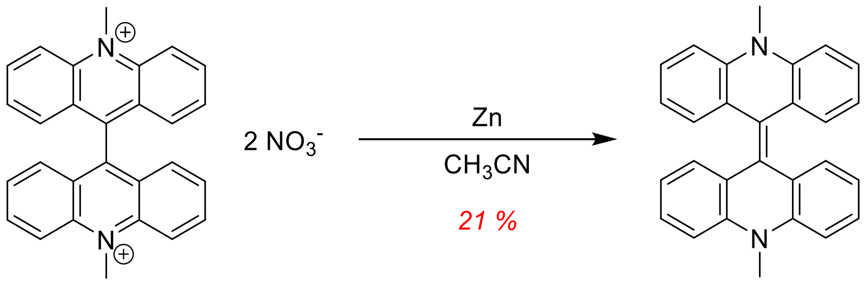

50 mg Lucigenin (as nitrate salt, 0.98 mmol, 1 equiv.) was dissolved in 50 mL CH_3CN. To this 256 mg Zn powder (3.918 mmol, 40 equiv.) was added and the resulting suspension was sonicated for 1.5 h. Afterwards 150 mL DCM were added and the suspension filtered. The clear organic phase was then washed with water (3x 100 mL). After drying over $MgSO_4$ the solvent was removed in vacuo. The product was purified by recrystallisation from a 1:1

mixture of hexane and toluene. The product was then washed with a small amount of pentane, affording 8 mg (21%) of **DMBA** as a dark yellow solid.

^{1}H NMR (400 MHz, $CDCl_3$) δ 7.17 (ddd, *J* = 8.6, 7.3, 1.6 Hz, 4H), 7.02 (td, *J* = 8.2, 1.3 Hz, 8H), 6.72 (ddd, *J* = 7.9, 7.3, 1.1 Hz, 4H), 3.53 (s, 6H).

This data is in accordance with literature.[3-4]

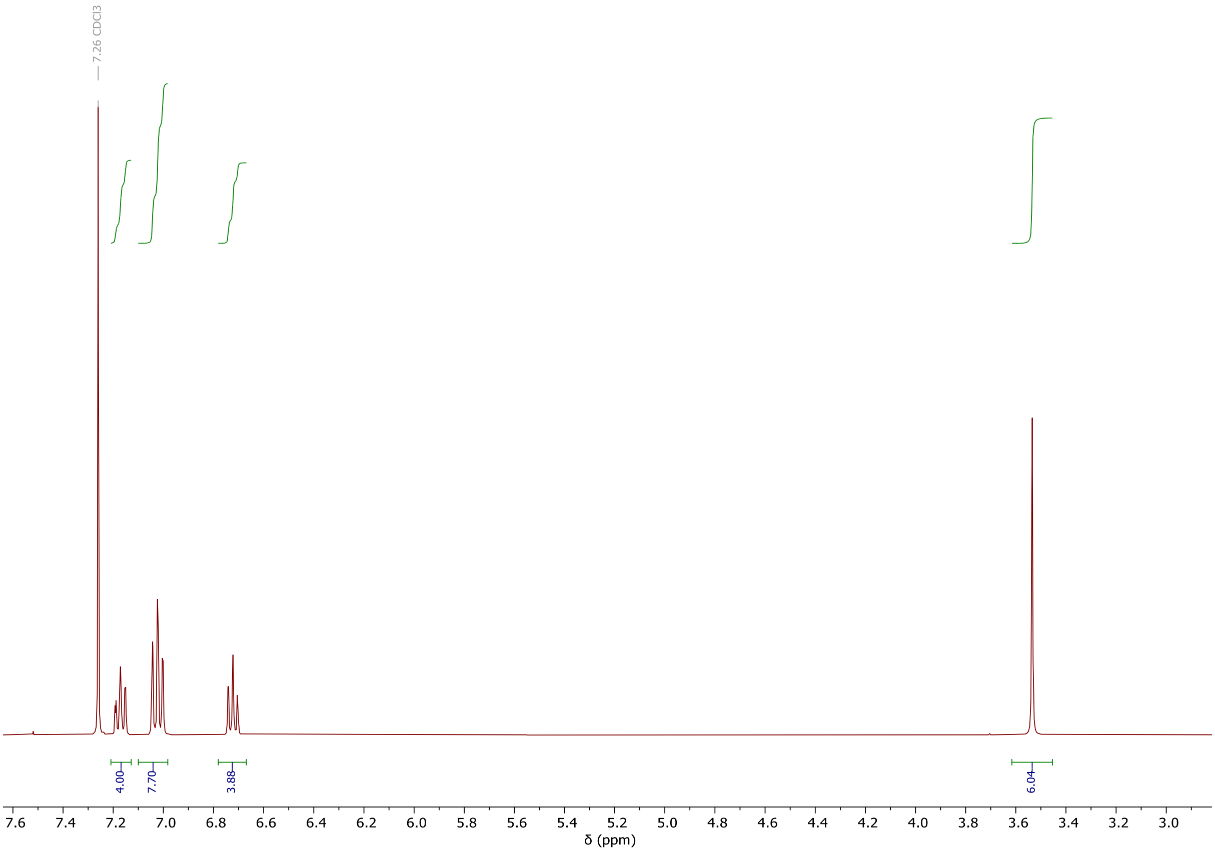

Figure S7. ^{1}H NMR (400 MHz, $CDCl_3$) of **DMBA**.

3. X-ray structural data

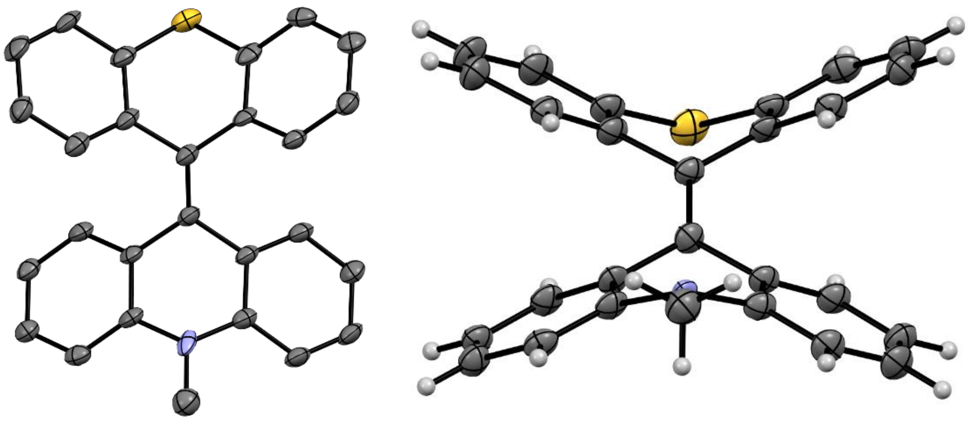

Figure S8. Front-view (left) and bottom-view (right) ORTEP image of neutral **TX-Acr**. Ellipsoids are drawn at 50% probability, and hydrogens are omitted for clarity.

Crystals of the neutral **TX-Acr** suitable for X-ray diffraction were obtained by layering toluene on top of a solution of **TX-Acr** in DCM. A single crystal was mounted on a cryoloop and placed in the nitrogen stream (100 K) of a Bruker-AXS D8 Venture diffractometer, using Cu Kα radiation (λ = 1.54178 Å). The Bruker APEX4 software suite was used for data collection and processing, and a multi-scan absorption correction was applied, based on the intensities of symmetry-related reflections measured at different angular settings (SADABS-2016/2).[5] The structure was solved using SHELXT.[6] Subsequent refinement was done using SHELXL[7] in the OLEX2 software package.[8] Hydrogen atoms were generated by geometrical considerations and refined using a riding model. No A- or B-level alerts were raised by CheckCIF for the fully refined structure.

Table S1. Crystallographic data for TX-Acr.

Empirical formula	$C_{27}H_{19}NS$
Formula weight	389.49
Temperature/K	100.00
Crystal system	monoclinic
Space group	$P2_1$
a/Å	7.2790(6)
b/Å	14.8462(11)
c/Å	8.9049(7)
α/°	90
β/°	98.648(5)
γ/°	90
Volume/Å^3	951.37(13)
Z	2
ρ_{calc}g/cm^3	1.360

μ/mm^{-1}	1.594
F(000)	408.0
Crystal size/mm^3	0.1 × 0.073 × 0.07
Radiation	CuKα (λ = 1.54178)
2Θ range for data collection/°	10.048 to 136.484
Index ranges	-8 ≤ h ≤ 8, -17 ≤ k ≤ 17, -10 ≤ l ≤ 10
Reflections collected	23377
Independent reflections	3452 [R_{int} = 0.1250, R_{sigma} = 0.0715]
Data/restraints/parameters	3452/1/263
Goodness-of-fit on F^2	1.043
Final R indexes [I>=2σ (I)]	R_1 = 0.0556, wR_2 = 0.1322
Final R indexes [all data]	R_1 = 0.0665, wR_2 = 0.1400
Largest diff. peak/hole / e Å^{-3}	0.34/-0.37
Flack parameter	0.122(19)

Table S2. Bond Lengths for TX-Acr.

Atom	Atom	Length/Å	Atom	Atom	Length/Å
S1	C7	1.764(5)	C12	C7	1.405(7)
S1	C1	1.759(6)	C25	C24	1.364(8)
N1	C26	1.448(7)	C17	C16	1.385(8)
N1	C20	1.455(6)	C15	C20	1.400(7)
N1	C27	1.368(7)	C15	C16	1.396(7)
C13	C14	1.359(6)	C6	C5	1.395(8)
C13	C12	1.488(7)	C6	C1	1.403(7)
C13	C6	1.492(7)	C22	C23	1.387(7)
C26	C21	1.407(7)	C20	C19	1.397(8)
C26	C25	1.397(7)	C5	C4	1.403(7)
C14	C21	1.491(7)	C2	C1	1.405(7)
C14	C15	1.489(7)	C2	C3	1.380(9)
C18	C17	1.382(7)	C9	C8	1.391(8)
C18	C19	1.387(8)	C9	C10	1.381(8)
C21	C22	1.393(8)	C24	C23	1.384(8)
C11	C12	1.394(7)	C4	C3	1.393(9)
C11	C10	1.388(8)	C7	C8	1.389(8)

Table S3. Bond Angles for TX-Acr.

Atom	Atom	Atom	Angle/°	Atom	Atom	Atom	Angle/°
C1	S1	C7	97.7(3)	C16	C15	C20	119.0(5)
C26	N1	C20	111.6(4)	C5	C6	C13	122.2(5)
C27	N1	C26	120.0(4)	C5	C6	C1	119.0(4)
C27	N1	C20	121.2(5)	C1	C6	C13	118.7(5)
C14	C13	C12	124.0(4)	C23	C22	C21	120.3(5)
C14	C13	C6	123.2(5)	C15	C20	N1	119.0(5)
C12	C13	C6	112.8(4)	C19	C20	N1	121.4(4)

C21	C26	N1	119.3(4)	C19	C20	C15	119.6(5)
C25	C26	N1	121.7(4)	C17	C16	C15	120.7(5)
C25	C26	C21	119.0(5)	C18	C19	C20	120.2(5)
C13	C14	C21	125.8(4)	C6	C5	C4	120.9(5)
C13	C14	C15	124.1(4)	C3	C2	C1	120.8(5)
C15	C14	C21	110.1(4)	C10	C9	C8	120.6(5)
C17	C18	C19	120.1(5)	C25	C24	C23	120.7(5)
C26	C21	C14	117.0(4)	C3	C4	C5	119.6(6)
C22	C21	C26	119.4(4)	C12	C7	S1	120.8(4)
C22	C21	C14	123.4(4)	C8	C7	S1	118.1(4)
C10	C11	C12	121.7(5)	C8	C7	C12	121.1(5)
C11	C12	C13	122.9(5)	C7	C8	C9	119.5(5)
C11	C12	C7	117.7(5)	C9	C10	C11	119.5(5)
C7	C12	C13	119.4(4)	C24	C23	C22	119.8(5)
C24	C25	C26	120.8(5)	C6	C1	S1	121.6(4)
C18	C17	C16	120.0(5)	C6	C1	C2	119.6(5)
C20	C15	C14	117.5(4)	C2	C1	S1	118.8(4)
C16	C15	C14	123.3(5)	C2	C3	C4	120.0(5)

Table S4. Dihedral Angles for TX-Acr.

A	B	C	D	Angle/˚	A	B	C	D	Angle/˚
S1	C7	C8	C9	179.2(4)	C12	C7	C8	C9	-1.4(8)
N1	C26	C21	C14	0.6(7)	C25	C26	C21	C14	178.8(5)
N1	C26	C21	C22	-174.9(5)	C25	C26	C21	C22	3.3(8)
N1	C26	C25	C24	176.5(5)	C25	C24	C23	C22	0.2(9)
N1	C20	C19	C18	-175.5(5)	C17	C18	C19	C20	-0.2(8)
C13	C14	C21	C26	139.6(5)	C15	C14	C21	C26	-39.5(6)
C13	C14	C21	C22	-45.1(8)	C15	C14	C21	C22	135.8(5)
C13	C14	C15	C20	-138.7(5)	C15	C20	C19	C18	3.3(8)
C13	C14	C15	C16	46.2(8)	C6	C13	C14	C21	-178.4(5)
C13	C12	C7	S1	0.1(7)	C6	C13	C14	C15	0.5(8)
C13	C12	C7	C8	-179.3(5)	C6	C13	C12	C11	131.8(5)
C13	C6	C5	C4	-180.0(5)	C6	C13	C12	C7	-45.1(7)
C13	C6	C1	S1	0.8(7)	C6	C5	C4	C3	-1.8(8)
C13	C6	C1	C2	-179.1(5)	C20	N1	C26	C21	38.3(6)
C26	N1	C20	C15	-37.4(6)	C20	N1	C26	C25	-139.9(5)
C26	N1	C20	C19	141.3(5)	C20	C15	C16	C17	5.0(8)
C26	C21	C22	C23	-3.2(8)	C16	C15	C20	N1	173.1(5)
C26	C25	C24	C23	-0.1(9)	C16	C15	C20	C19	-5.7(7)
C14	C13	C12	C11	-47.7(8)	C19	C18	C17	C16	-0.6(8)
C14	C13	C12	C7	135.4(5)	C5	C6	C1	S1	177.1(4)
C14	C13	C6	C5	47.8(8)	C5	C6	C1	C2	-2.9(8)

C14	C13	C6	C1	-136.0(5)	C5	C4	C3	C2	-1.4(9)
C14	C21	C22	C23	-178.5(5)	C7	S1	C1	C6	-37.2(5)
C14	C15	C20	N1	-2.2(7)	C7	S1	C1	C2	142.7(4)
C14	C15	C20	C19	179.0(5)	C8	C9	C10	C11	1.3(8)
C14	C15	C16	C17	-180.0(5)	C10	C11	C12	C13	179.5(5)
C18	C17	C16	C15	-1.9(8)	C10	C11	C12	C7	-3.5(8)
C21	C26	C25	C24	-1.7(8)	C10	C9	C8	C7	-1.1(8)
C21	C14	C15	C20	40.4(6)	C1	S1	C7	C12	36.6(5)
C21	C14	C15	C16	-134.7(5)	C1	S1	C7	C8	-144.0(4)
C21	C22	C23	C24	1.5(8)	C1	C6	C5	C4	3.9(8)
C11	C12	C7	S1	-176.9(4)	C1	C2	C3	C4	2.4(9)
C11	C12	C7	C8	3.6(8)	C3	C2	C1	S1	179.8(5)
C12	C13	C14	C21	1.1(8)	C3	C2	C1	C6	-0.3(8)
C12	C13	C14	C15	-180.0(5)	C27	N1	C26	C21	-171.1(5)
C12	C13	C6	C5	-131.7(5)	C27	N1	C26	C25	10.8(8)
C12	C13	C6	C1	44.5(7)	C27	N1	C20	C15	172.3(5)
C12	C11	C10	C9	1.1(8)	C27	N1	C20	C19	-8.9(7)

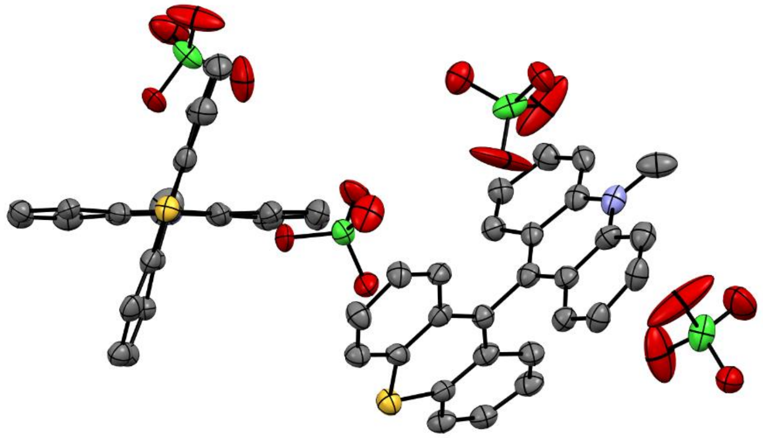

Figure S9. ORTEP image of **TX-Acr^{2+}(ClO_4^-)$_2$**. Ellipsoids are drawn at 50% probability, and hydrogens are omitted for clarity.

Crystals of the dicationic **TX-Acr^{2+}(ClO_4^-)$_2$** suitable for X-ray diffraction were obtained by vapour diffusion of pentane into a solution of **TX-Acr^{2+}(ClO_4^-)$_2$** in CH_3CN. A single crystal was mounted on a cryoloop and placed in the nitrogen stream (100 K) of a Bruker-AXS D8 Venture diffractometer, using Cu Kα radiation (λ = 1.54178 Å). The Bruker APEX4 software suite was used for data collection and processing, and a multi-scan absorption correction was applied, based on the intensities of symmetry-related reflections measured at different angular settings (SADABS-2016/2).[5] The structure was solved using SHELXT.[6] Subsequent refinement was done using SHELXL[7] in the OLEX2 software package.[8] Hydrogen atoms were generated by geometrical considerations and refined using a riding model. Two molecules of the **TX-**

Acr^{2+}occupy one unit cell. One of the two molecules in the unit cell was found to adopt two different orientations in the crystal, in which the positions of the NCH_3 and S groups are opposite. The two partial geometry orientations of the S and NCH_3 moieties were modelled at occupancies of 25% and 75%. One B-level alert was raised for the fully refined structure for low bond precision on C-C bonds. Possibly, this is partially due to slight disorder of the remaining atoms that were not separately modelled in the two different orientations. Multiple crystallizations were performed, but the various crystallization methods did not lead to higher-quality data.

Table S5. Crystallographic data for TX-Acr^{2+}(ClO_4^-)$_2$.

Empirical formula	$C_{27}H_{19}Cl_2NO_8S$
Formula weight	588.39
Temperature/K	100.00
Crystal system	monoclinic
Space group	$P2_1/c$
a/Å	15.9085(6)
b/Å	18.9168(8)
c/Å	16.4929(6)
α/°	90
β/°	95.412(3)
γ/°	90
Volume/Å^3	4941.2(3)
Z	8
ρ_{calc}g/cm^3	1.582
μ/mm^{-1}	3.642
F(000)	2416.0
Crystal size/mm^3	0.073 × 0.05 × 0.013
Radiation	CuKα (λ = 1.54178)
2Θ range for data collection/°	5.58 to 136.48
Index ranges	$-16 \le h \le 19$, $-22 \le k \le 22$, $-19 \le l \le 19$
Reflections collected	150171
Independent reflections	9038 [R_{int} = 0.1491, R_{sigma} = 0.0421]
Data/restraints/parameters	9038/0/733
Goodness-of-fit on F^2	1.028
Final R indexes [I>=2σ (I)]	R_1 = 0.1218, wR_2 = 0.2477
Final R indexes [all data]	R_1 = 0.1423, wR_2 = 0.2607
Largest diff. peak/hole / e Å^{-3}	1.20/-0.94

The following A- and B-level ALERTS were generated:

Alert level B

PLAT340_ALERT_3_B Low Bond Precision on C-C Bonds .... 0.01122 Ang.

This is likely partially a result of remaining disorder of atoms that were not explicitly modelled in the two possible orientations that the molecule can adopt in the crystal, as this was only done for the S and NCH_3 moieties. Several attempts were made to grow better crystals but no higher-quality data was obtained.

Table S6. Bond Lengths for TX-$Acr^{2+}(ClO_4^-)_2$.

Atom	Atom	Length/Å	Atom	Atom	Length/Å
Cl1C	O4C	1.446(5)	C12A	C11A	1.428(10)
Cl1C	O1C	1.445(5)	C12A	C13A	1.400(10)
Cl1C	O3C	1.426(6)	C12A	C7A	1.419(10)
Cl1C	O2C	1.434(6)	C8A	C9A	1.361(11)
Cl1B	O1B	1.441(6)	C8A	C7A	1.410(11)
Cl1B	O2B	1.444(6)	C16A	C17A	1.365(11)
Cl1B	O4B	1.443(8)	C2A	C1A	1.412(10)
Cl1B	O3B	1.393(7)	C2A	C3A	1.368(11)
Cl1D	O4D	1.430(5)	C15B	C16B	1.429(11)
Cl1D	O1D	1.417(7)	C15B	C20B	1.424(12)
Cl1D	O3D	1.485(8)	C23B	C24B	1.403(11)
Cl1D	O2D	1.408(7)	C13A	C6A	1.433(10)
S1A	C1A	1.714(8)	C21B	C26B	1.412(11)
S1A	C7A	1.701(8)	C16B	C17B	1.357(11)
Cl1A	O3A	1.435(6)	C17A	C18A	1.399(11)
Cl1A	O1A	1.454(7)	C10B	C11B	1.380(11)
Cl1A	O4A	1.449(8)	C10B	C9B	1.388(11)
Cl1A	O2A	1.381(8)	C5B	C4B	1.380(10)
S1B	C7B	1.742(9)	C24B	C25B	1.370(11)
S1B	C1B	1.701(8)	C5A	C6A	1.445(11)
N1A	C26A	1.373(10)	C5A	C4A	1.356(11)
N1A	C20A	1.391(10)	C26B	C25B	1.427(12)
N1A	C27A	1.360(12)	C26B	S1C	1.795(14)
C15A	C14A	1.386(11)	C26B	N1B	1.383(13)
C15A	C16A	1.431(10)	C20A	C19A	1.396(11)
C15A	C20A	1.425(11)	C19A	C18A	1.384(12)
C13B	C12B	1.425(10)	C1A	C6A	1.413(11)
C13B	C6B	1.397(11)	C9B	C8B	1.375(11)
C13B	C14B	1.486(11)	C8B	C7B	1.403(11)
C22B	C23B	1.363(10)	C7B	N1C	1.36(3)

C22B	C21B	1.440(10)
C12B	C11B	1.427(10)
C12B	C7B	1.416(11)
C10A	C11A	1.378(11)
C10A	C9A	1.406(11)
C6B	C5B	1.442(11)
C6B	C1B	1.445(11)
C14B	C15B	1.411(10)
C14B	C21B	1.426(11)
C14A	C21A	1.408(10)
C14A	C13A	1.506(10)
C26A	C21A	1.418(11)
C26A	C25A	1.435(11)
C22A	C21A	1.442(11)
C22A	C23A	1.356(11)
C20B	C19B	1.417(12)
C20B	S1C	1.823(15)
C20B	N1B	1.348(14)
C4B	C3B	1.390(12)
C18B	C17B	1.402(12)
C18B	C19B	1.372(12)
C1B	C2B	1.404(12)
C1B	N1C	1.41(3)
C4A	C3A	1.419(12)
C2B	C3B	1.367(12)
C23A	C24A	1.421(12)
C25A	C24A	1.339(12)
C27B	N1B	1.498(14)
C1C	N1C	1.55(5)

Table S7. Bond Angles for TX-Acr^{2+}(ClO_4^-)$_2$.

Atom	Atom	Atom	Angle/˚
O1C	Cl1C	O4C	109.2(3)
O3C	Cl1C	O4C	109.0(4)
O3C	Cl1C	O1C	111.7(4)
O3C	Cl1C	O2C	109.9(4)
O2C	Cl1C	O4C	106.7(4)
O2C	Cl1C	O1C	110.3(4)
O1B	Cl1B	O2B	109.5(4)
O1B	Cl1B	O4B	108.5(4)
O4B	Cl1B	O2B	106.4(6)
O3B	Cl1B	O1B	111.8(6)
O3B	Cl1B	O2B	107.7(4)
O3B	Cl1B	O4B	112.7(8)
O4D	Cl1D	O3D	104.6(4)
O1D	Cl1D	O4D	111.2(4)
O1D	Cl1D	O3D	107.6(5)
O2D	Cl1D	O4D	112.0(4)
O2D	Cl1D	O1D	114.6(5)
O2D	Cl1D	O3D	106.1(6)
C7A	S1A	C1A	103.3(4)
O3A	Cl1A	O1A	108.3(4)
O3A	Cl1A	O4A	107.6(5)
C12A	C13A	C6A	124.2(7)
C6A	C13A	C14A	115.8(7)
C14B	C21B	C22B	120.4(7)
C26B	C21B	C22B	119.3(7)
C26B	C21B	C14B	120.2(7)
C17B	C16B	C15B	120.3(8)
C16A	C17A	C18A	119.5(8)
C11B	C10B	C9B	121.0(8)
C4B	C5B	C6B	119.7(8)
C25B	C24B	C23B	121.0(8)
C4A	C5A	C6A	120.3(8)
C10B	C11B	C12B	119.8(8)
C21B	C26B	C25B	119.8(7)
C21B	C26B	S1C	137.9(8)
C25B	C26B	S1C	102.2(7)
N1B	C26B	C21B	115.5(9)
N1B	C26B	C25B	124.7(9)
N1A	C20A	C15A	118.8(7)
N1A	C20A	C19A	120.7(7)
C19A	C20A	C15A	120.4(7)
C18A	C19A	C20A	120.0(8)

O4A	Cl1A	O1A	105.9(5)
O2A	Cl1A	O3A	110.0(5)
O2A	Cl1A	O1A	112.6(8)
O2A	Cl1A	O4A	112.3(8)
C1B	S1B	C7B	100.1(4)
C26A	N1A	C20A	121.9(7)
C27A	N1A	C26A	121.6(7)
C27A	N1A	C20A	116.6(8)
C14A	C15A	C16A	123.0(7)
C14A	C15A	C20A	119.8(7)
C20A	C15A	C16A	117.1(7)
C12B	C13B	C14B	117.6(7)
C6B	C13B	C12B	123.1(7)
C6B	C13B	C14B	119.3(7)
C23B	C22B	C21B	118.7(7)
C13B	C12B	C11B	120.8(7)
C7B	C12B	C13B	121.5(7)
C7B	C12B	C11B	117.7(7)
C11A	C10A	C9A	120.5(7)
C13B	C6B	C5B	121.0(8)
C13B	C6B	C1B	121.5(7)
C5B	C6B	C1B	117.4(7)
C15B	C14B	C13B	121.7(7)
C15B	C14B	C21B	119.3(8)
C21B	C14B	C13B	119.0(7)
C15A	C14A	C21A	120.1(7)
C15A	C14A	C13A	119.8(7)
C21A	C14A	C13A	120.1(7)
N1A	C26A	C21A	119.3(7)
N1A	C26A	C25A	121.8(8)
C21A	C26A	C25A	118.9(7)
C23A	C22A	C21A	120.9(8)
C13A	C12A	C11A	119.8(7)
C13A	C12A	C7A	121.5(7)
C7A	C12A	C11A	118.8(7)
C14A	C21A	C26A	119.6(7)
C14A	C21A	C22A	122.1(7)
C26A	C21A	C22A	118.3(7)
C10A	C11A	C12A	119.4(7)
C9A	C8A	C7A	119.2(7)
C17A	C16A	C15A	121.8(7)
C19A	C18A	C17A	121.0(8)
C2A	C1A	S1A	114.5(6)
C2A	C1A	C6A	121.0(7)
C6A	C1A	S1A	124.4(6)
C8B	C9B	C10B	121.2(8)
C9B	C8B	C7B	118.9(8)
C8A	C9A	C10A	121.6(8)
C12B	C7B	S1B	126.3(6)
C8B	C7B	S1B	112.3(6)
C8B	C7B	C12B	121.3(7)
N1C	C7B	C12B	104.2(17)
N1C	C7B	C8B	134.3(18)
C15B	C20B	S1C	135.9(8)
C19B	C20B	C15B	120.0(8)
C19B	C20B	S1C	104.0(8)
N1B	C20B	C15B	115.2(9)
N1B	C20B	C19B	124.8(9)
C13A	C6A	C5A	121.7(7)
C1A	C6A	C13A	121.0(7)
C1A	C6A	C5A	117.2(7)
C5B	C4B	C3B	121.0(8)
C19B	C18B	C17B	122.1(8)
C6B	C1B	S1B	126.9(6)
C2B	C1B	S1B	112.8(6)
C2B	C1B	C6B	120.3(7)
C2B	C1B	N1C	136.3(16)
N1C	C1B	C6B	103.3(16)
C12A	C7A	S1A	124.8(6)
C8A	C7A	S1A	114.8(6)
C8A	C7A	C12A	120.3(7)
C5A	C4A	C3A	121.4(8)
C3B	C2B	C1B	119.8(8)
C24B	C25B	C26B	119.1(8)
C22A	C23A	C24A	119.3(8)
C2B	C3B	C4B	121.7(8)
C16B	C17B	C18B	120.3(8)
C18B	C19B	C20B	118.5(9)
C24A	C25A	C26A	120.0(9)
C25A	C24A	C23A	122.2(8)
C2A	C3A	C4A	119.8(8)
C26B	S1C	C20B	85.7(8)

C3A	C2A	C1A	119.9(8)	C26B	N1B	C27B	113.9(11)
C14B	C15B	C16B	120.4(7)	C20B	N1B	C26B	128.6(11)
C14B	C15B	C20B	121.0(7)	C20B	N1B	C27B	117.4(11)
C20B	C15B	C16B	118.5(7)	C7B	N1C	C1B	145(3)
C22B	C23B	C24B	121.9(8)	C7B	N1C	C1C	104(3)
C12A	C13A	C14A	120.0(7)	C1B	N1C	C1C	111(3)

Table S8. Dihedral Angles for TX-Acr^{2+}(ClO_4^-)$_2$.

A	B	C	D	Angle/˚	A	B	C	D	Angle/˚
S1A	C1A	C6A	C13A	-9.9(11)	C23B	C22B	C21B	C14B	-178.9(7)
S1A	C1A	C6A	C5A	174.5(6)	C23B	C22B	C21B	C26B	4.0(10)
S1B	C1B	C2B	C3B	-178.8(7)	C23B	C24B	C25B	C26B	0.2(12)
N1A	C26A	C21A	C14A	-5.9(11)	C13A	C14A	C21A	C26A	-178.7(7)
N1A	C26A	C21A	C22A	174.6(7)	C13A	C14A	C21A	C22A	0.7(11)
N1A	C26A	C25A	C24A	-178.6(8)	C13A	C12A	C11A	C10A	-175.5(7)
N1A	C20A	C19A	C18A	179.9(7)	C13A	C12A	C7A	S1A	-8.1(11)
C15A	C14A	C21A	C26A	0.5(11)	C13A	C12A	C7A	C8A	174.8(7)
C15A	C14A	C21A	C22A	179.9(7)	C21B	C22B	C23B	C24B	0.2(11)
C15A	C14A	C13A	C12A	-108.3(8)	C21B	C14B	C15B	C16B	179.8(7)
C15A	C14A	C13A	C6A	68.5(9)	C21B	C14B	C15B	C20B	2.4(11)
C15A	C16A	C17A	C18A	-0.7(12)	C21B	C26B	C25B	C24B	3.9(11)
C15A	C20A	C19A	C18A	-1.1(12)	C21B	C26B	S1C	C20B	-2.1(11)
C13B	C12B	C11B	C10B	-174.8(7)	C21B	C26B	N1B	C20B	6.8(14)
C13B	C12B	C7B	S1B	-7.8(10)	C21B	C26B	N1B	C27B	-171.1(9)
C13B	C12B	C7B	C8B	173.8(7)	C16B	C15B	C20B	C19B	3.1(11)
C13B	C12B	C7B	N1C	-9.8(15)	C16B	C15B	C20B	S1C	178.7(8)
C13B	C6B	C5B	C4B	-175.0(7)	C16B	C15B	C20B	N1B	-176.4(8)
C13B	C6B	C1B	S1B	-6.3(11)	C10B	C9B	C8B	C7B	0.2(11)
C13B	C6B	C1B	C2B	175.5(7)	C5B	C6B	C1B	S1B	175.8(6)
C13B	C6B	C1B	N1C	-1.7(14)	C5B	C6B	C1B	C2B	-2.4(11)
C13B	C14B	C15B	C16B	-3.0(11)	C5B	C6B	C1B	N1C	-179.5(13)
C13B	C14B	C15B	C20B	179.7(7)	C5B	C4B	C3B	C2B	-2.2(13)
C13B	C14B	C21B	C22B	4.0(10)	C5A	C4A	C3A	C2A	-3.3(12)
C13B	C14B	C21B	C26B	-178.9(7)	C11B	C12B	C7B	S1B	173.7(6)
C22B	C23B	C24B	C25B	-2.3(12)	C11B	C12B	C7B	C8B	-4.8(10)
C22B	C21B	C26B	C25B	-6.0(11)	C11B	C12B	C7B	N1C	171.7(13)
C22B	C21B	C26B	S1C	179.1(8)	C11B	C10B	C9B	C8B	-1.1(12)
C22B	C21B	C26B	N1B	174.6(7)	C20A	N1A	C26A	C21A	5.4(11)
C12B	C13B	C6B	C5B	179.4(7)	C20A	N1A	C26A	C25A	-173.7(7)

C12B	C13B	C6B	C1B	1.6(11)
C12B	C13B	C14B	C15B	-103.6(8)
C12B	C13B	C14B	C21B	73.6(9)
C12B	C7B	N1C	C1B	14(4)
C12B	C7B	N1C	C1C	-173(3)
C6B	C13B	C12B	C11B	-176.2(7)
C6B	C13B	C12B	C7B	5.2(11)
C6B	C13B	C14B	C15B	75.5(9)
C6B	C13B	C14B	C21B	-107.2(8)
C6B	C5B	C4B	C3B	-0.6(12)
C6B	C1B	C2B	C3B	-0.3(12)
C6B	C1B	N1C	C7B	-8(4)
C6B	C1B	N1C	C1C	179(3)
C14B	C13B	C12B	C11B	2.9(10)
C14B	C13B	C12B	C7B	-175.6(7)
C14B	C13B	C6B	C5B	0.3(11)
C14B	C13B	C6B	C1B	-177.5(7)
C14B	C15B	C16B	C17B	-177.2(7)
C14B	C15B	C20B	C19B	-179.5(7)
C14B	C15B	C20B	S1C	-3.9(13)
C14B	C15B	C20B	N1B	1.0(11)
C14B	C21B	C26B	C25B	176.9(7)
C14B	C21B	C26B	S1C	2.0(13)
C14B	C21B	C26B	N1B	-2.5(11)
C14A	C15A	C16A	C17A	-174.1(7)
C14A	C15A	C20A	N1A	-5.9(11)
C14A	C15A	C20A	C19A	175.1(7)
C14A	C13A	C6A	C5A	2.7(11)
C14A	C13A	C6A	C1A	-172.7(7)
C26A	N1A	C20A	C15A	0.4(11)
C26A	N1A	C20A	C19A	179.4(7)
C26A	C25A	C24A	C23A	2.9(13)
C22A	C23A	C24A	C25A	-3.9(13)
C12A	C13A	C6A	C5A	179.4(7)
C12A	C13A	C6A	C1A	4.0(12)
C21A	C14A	C13A	C12A	70.9(9)
C21A	C14A	C13A	C6A	-112.3(8)
C21A	C26A	C25A	C24A	2.3(12)
C21A	C22A	C23A	C24A	-0.3(12)
C11A	C10A	C9A	C8A	-0.8(13)
C11A	C12A	C13A	C14A	0.8(11)
C20A	C15A	C14A	C21A	5.4(11)
C20A	C15A	C14A	C13A	-175.4(7)
C20A	C15A	C16A	C17A	3.2(11)
C20A	C19A	C18A	C17A	3.7(12)
C1A	S1A	C7A	C12A	2.5(8)
C1A	S1A	C7A	C8A	179.7(6)
C1A	C2A	C3A	C4A	2.8(12)
C9B	C10B	C11B	C12B	-1.0(11)
C9B	C8B	C7B	S1B	-175.8(6)
C9B	C8B	C7B	C12B	2.8(11)
C9B	C8B	C7B	N1C	-172.4(18)
C8B	C7B	N1C	C1B	-171(3)
C8B	C7B	N1C	C1C	2(4)
C9A	C10A	C11A	C12A	-1.1(12)
C9A	C8A	C7A	S1A	-174.9(6)
C9A	C8A	C7A	C12A	2.4(12)
C7B	S1B	C1B	C6B	3.6(8)
C7B	S1B	C1B	C2B	-178.0(6)
C7B	C12B	C11B	C10B	3.8(10)
C20B	C15B	C16B	C17B	0.2(11)
C6A	C5A	C4A	C3A	-1.1(12)
C1B	S1B	C7B	C12B	3.2(7)
C1B	S1B	C7B	C8B	-178.2(6)
C1B	C6B	C5B	C4B	2.8(11)
C1B	C2B	C3B	C4B	2.7(13)
C7A	S1A	C1A	C2A	-172.8(6)
C7A	S1A	C1A	C6A	6.3(7)
C7A	C12A	C11A	C10A	3.6(11)
C7A	C12A	C13A	C14A	-178.3(7)
C7A	C12A	C13A	C6A	5.2(12)
C7A	C8A	C9A	C10A	0.1(13)
C4A	C5A	C6A	C13A	-169.7(7)
C4A	C5A	C6A	C1A	5.9(11)
C2B	C1B	N1C	C7B	176(3)
C2B	C1B	N1C	C1C	3(4)
C25B	C26B	S1C	C20B	-177.6(6)
C25B	C26B	N1B	C20B	-172.6(9)
C25B	C26B	N1B	C27B	9.5(14)
C23A	C22A	C21A	C14A	-174.1(7)
C23A	C22A	C21A	C26A	5.3(11)
C17B	C18B	C19B	C20B	1.1(13)

C11A	C12A	C13A	C6A	-175.8(7)	C27A	N1A	C26A	C21A	-172.4(8)
C11A	C12A	C7A	S1A	172.8(6)	C27A	N1A	C26A	C25A	8.5(12)
C11A	C12A	C7A	C8A	-4.3(11)	C27A	N1A	C20A	C15A	178.4(8)
C16A	C15A	C14A	C21A	-177.4(7)	C27A	N1A	C20A	C19A	-2.6(11)
C16A	C15A	C14A	C13A	1.7(11)	C19B	C20B	S1C	C26B	179.2(6)
C16A	C15A	C20A	N1A	176.8(7)	C19B	C20B	N1B	C26B	174.5(9)
C16A	C15A	C20A	C19A	-2.3(11)	C19B	C20B	N1B	C27B	-7.6(15)
C16A	C17A	C18A	C19A	-2.8(13)	C19B	C18B	C17B	C16B	2.2(13)
C2A	C1A	C6A	C13A	169.2(7)	C25A	C26A	C21A	C14A	173.2(7)
C2A	C1A	C6A	C5A	-6.4(11)	C25A	C26A	C21A	C22A	-6.2(11)
C15B	C14B	C21B	C22B	-178.7(6)	C3A	C2A	C1A	S1A	-178.6(6)
C15B	C14B	C21B	C26B	-1.6(10)	C3A	C2A	C1A	C6A	2.2(11)
C15B	C16B	C17B	C18B	-2.8(12)	S1C	C26B	C25B	C24B	-179.6(7)
C15B	C20B	C19B	C18B	-3.7(12)	S1C	C20B	C19B	C18B	179.4(7)
C15B	C20B	S1C	C26B	3.1(10)	N1B	C26B	C25B	C24B	-176.7(8)
C15B	C20B	N1B	C26B	-6.0(14)	N1B	C20B	C19B	C18B	175.7(9)
C15B	C20B	N1B	C27B	171.9(10)	N1C	C1B	C2B	C3B	175.6(18)

4. Computational analysis

Conformational Analysis

DFT calculations were performed using the Orca 5.0.4 package.[9] The geometries of the *anti*-folded, *syn*-folded and orthogonal/twisted conformations of the neutral, cationic and dicationic TX-Acr structures were optimized both with the r^2SCAN-3c[10] and the PBEh-3c[11] composite method. The thermochemical data were calculated at the same levels of theory. R^2SCAN-3c is a cost-efficient method that is a particularly appropriate choice for calculating conformer energies with high accuracy, while PBEh-3c, containing 42% Fock exchange, provides energies at a hybrid level of theory.[12] As can be seen from Table S9, both predict the same clear trend in relative stability of the various conformers. The counterions were not included in the calculations, and no solvent model was used. Very tight optimization convergence criteria were applied to the geometry optimizations. However, at the PBEh-3c level of theory, the neutral twisted state and dicationic *syn*-folded state could not be optimized as minima using these very tight optimization convergence criteria. Instead, they relaxed to the *anti*-folded and orthogonal state, respectively. However, optimization of the neutral twisted and *syn*-folded dicationic conformations was achieved by applying normal and loose optimization convergence criteria, respectively, which allowed to obtain approximate energies for these conformations. All obtained minima had no imaginary frequency.

Table S9. Computed energies (in kJ/mol) of the different conformations of **TX-Acr**, relative to the energy of the lowest conformation within each redox state. Calculations were performed at the r^2SCAN-3c and PBEh-3c levels of theory.

	**r^2-SCAN-3c**			**PBEh-3c**		
	**Neutral**	**Cationic**	**Dicationic**	**Neutral**	**Cationic**	**Dicationic**
**Anti-folded**	0	49	150	0	47	174
**Syn-folded**	39	97	257	38	91	309[b]
**Twisted**	52	0	-	78[a]	0	-
**Orthogonal**	-	-	0	-	-	0

a) normal optimization convergence criteria were used b) loose optimization convergence criteria were used

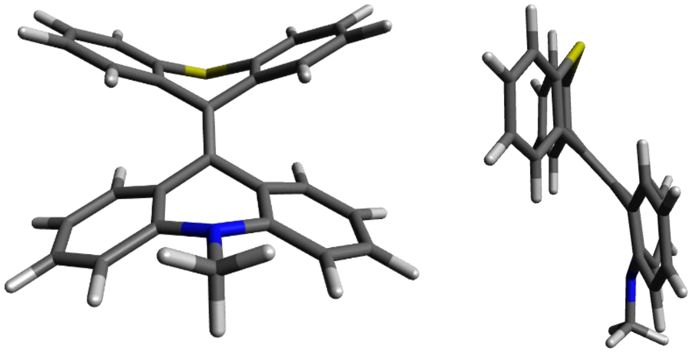

Figure S10. Calculated lowest-energy structure of **TX-Acr** in the *anti*-folded state at the r^2SCAN-3c level of theory (global minimum for this redox state).

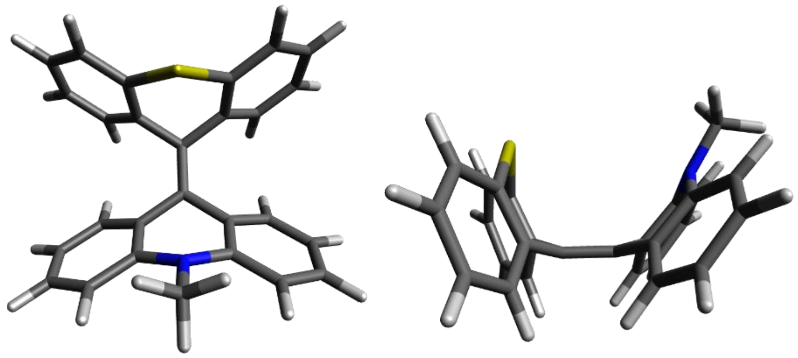

Figure S11. Calculated structure of **TX-Acr** in the *syn*-folded state at the r^2SCAN-3c level of theory (local minimum, metastable state).

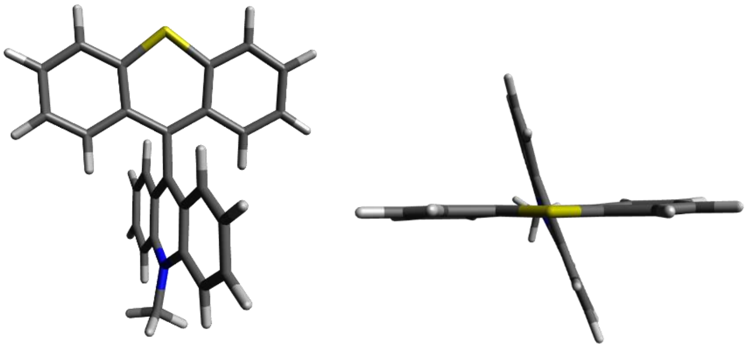

Figure S12. Calculated lowest energy structure of **TX-Acr**$^{+\bullet}$ in the twisted state at the r^2SCAN-3c level of theory (global minimum for this redox state).

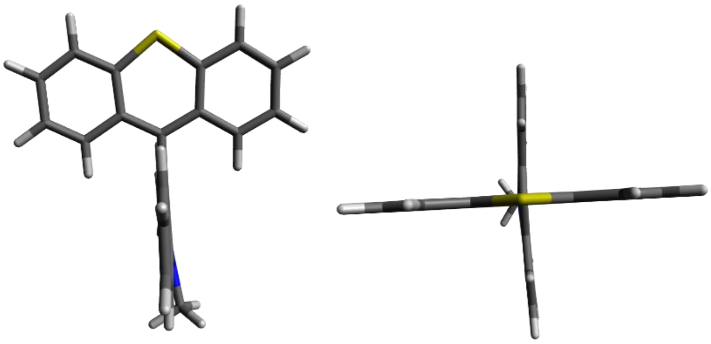

Figure S13. Calculated lowest energy structure of **TX-Acr**$^{2+}$ in the orthogonal state at the r^2SCAN-3c level of theory (global minimum for this redox state).

Thermal syn-anti relaxation

DFT calculations were performed to gain insight into the mechanism of the thermal relaxation from the syn-folded to the anti-folded conformation for all three switches and to explain the trend in the activation barriers of this step. DFT calculations were performed using the Orca 5.0.4 package[9] and were initially performed at the r^2SCAN-3c level of theory,[10] a cost-efficient composite method that has been recommended for obtaining reliable conformer energies. Very tight optimization criteria were used, and the thermochemical data were calculated at the same levels of theory. Single-point energies were calculated at the ωB97X-D4/def2-QZVP level of theory[13-16] to correct the electronic energy term in the computed Gibbs free energy

and obtain more accurate activation barriers. No solvent model was used. Minina and transition states had no or one imaginary frequency, respectively.

Table S10. Computed energies (in kJ/mol) of the different conformations and transition states of **TX-Acr**, **BTX** and **DMBA**, relative to the energy of their *syn*-folded conformation. Calculations were performed at the r^{2}SCAN-3c and ωB97X-D4/def2-QZVP//r^{2}SCAN-3c levels of theory.

	**r^{2}SCAN-3c**			**ωB97X-D4/def2-QZVP//r^{2}SCAN-3c**		
	**TX-Acr**	**BTX**	**DMBA**	**TX-Acr**	**BTX**	**DMBA**
Af	-38.7	-39.9	-41.1	-33.4	-34.2	-34.9
Syn	0.0	0.0	0.0	0.0	0.0	0.0
Tw	12.8	29.3	-8.4	65.4	90.0	44.5
**TS$_{Syn\text{-}Af}$**	51.5	83.7	59.3	59.8	95.2	70.8
**TS$_{Syn\text{-}Tw}$**	22.5	38.0	7.5	47.9	71.5	22.9
**TS$_{Tw\text{-}Af}$**	67.0	89.6	46.6 (54.9)[a]	96.4	124.3	80.7
**Experimental**	**55**	**82**[b]	**60**[b]	**55**	**82**[b]	**60**[b]

a) The value in parenthesis corresponds to the energy with respect to the twisted conformation, which directly precedes it and is calculated to be more stable than the *syn*-folded conformation.

b) Approximate barriers, calculated form literature values of the thermal half-life[17]

As can be seen from the results in Table S10, the energy difference between the *syn*-folded and *anti*-folded conformations is similar in all three overcrowded alkenes. Therefore, the difference in activation barrier is likely a result of a different degree of (de)stabilization in the transition state, rather than ground-state destabilization. Interestingly, the twisted conformation is much more destabilized in **BTX** than in **DMBA**, most likely because planarization of the nitrogen-containing structure is less unfavorable than planarization of the sulfur-containing structure, the latter strongly preferring a more folded conformation than the former. As can be seen in Figure S14, the transition states that directly connect the *syn*- and *antifolded* conformations (TS$_{Syn\text{-}Af}$) feature one half of the molecule in a folded geometry and the other half in a planarized geometry. This explains why **TX-Acr** has the relatively lowest transition state with respect to its *syn* folded conformer, as the mixed structure allows the **TX** part to adopt its preferred folded geometry and the **Acr** part to adopt the more planarized geometry.

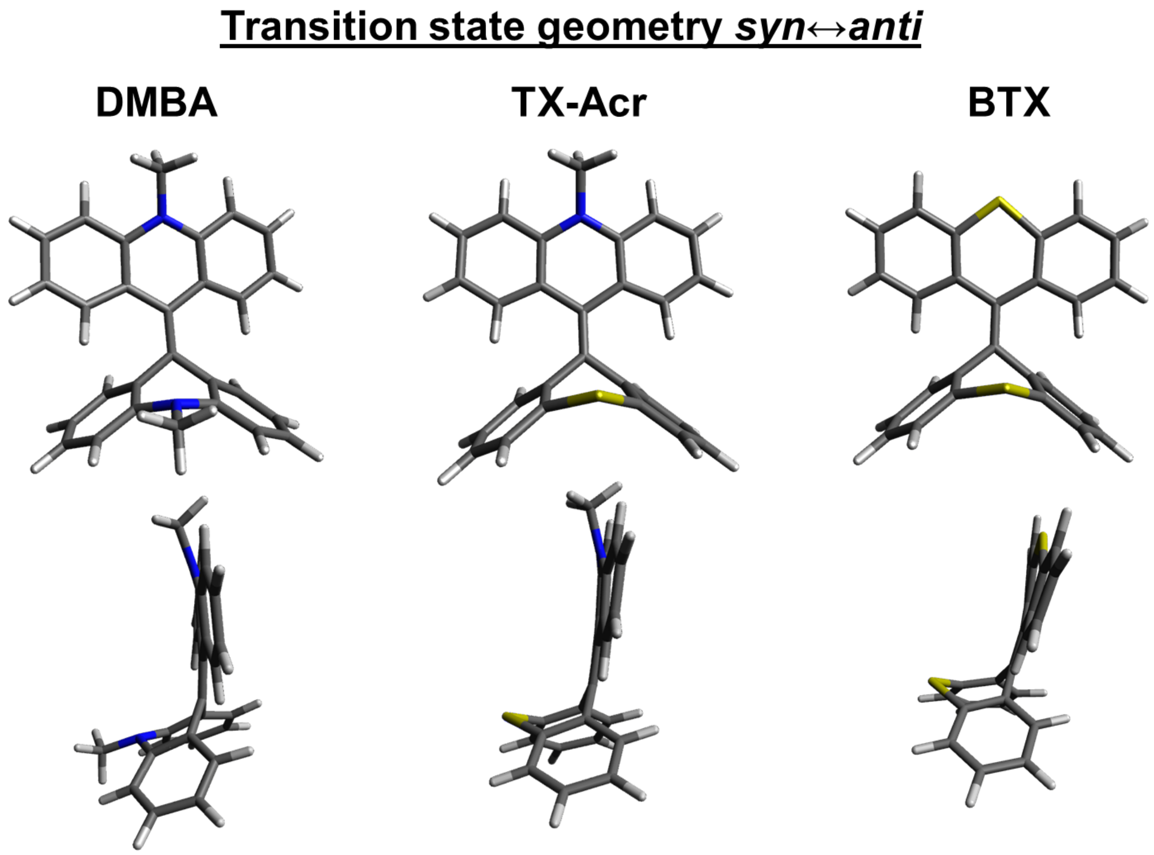

Figure S14. Calculated transition state structure at the r^{2}SCAN-3c level of theory for the *syn*- to *anti*-folded interconversion for all three switches.

It has to be noted that an alternative 2-step mechanism was found in which the *syn*-folded conformation first adopts a twisted conformation via $TS_{Syn\text{-}Tw}$ and subsequently an *anti*-folded conformation via $TS_{Tw\text{-}Af}$. This pathway was even calculated to be preferred over the direct *Syn-Anti* conversion for **DMBA** at the r^{2}SCAN-3c level of theory, with the twisted conformation calculated to be slightly more stable than the *syn*-folded conformation. However, experimentally the twisted conformation has not been observed as a photoproduct or intermediate on the relaxation pathway.[17-18] Therefore, the results at the higher ωB97X-D4/def2-QZVP//r^{2}SCAN-3c level of theory are in better agreement with the experimental observations. Of further note is that in a previous theoretical study that was done using the semi-empirical method PM3, the twisted conformation of **BTX** did not represent a minimum but rather a transition state and a structure similar to $TS_{Tw\text{-}Af}$ was found as a higher-order saddlepoint.[19]

The calculated values for the activation barriers are in good agreement with the experimentally found barriers and reproduce the trend between the different overcrowded alkenes well. Nevertheless, a difference of ~10 kJ/mol between the calculated and experimental barrier was found for **BTX** and **DMBA** at the ωB97X-D4/def2-QZVP//r^{2}SCAN-3c level of theory. This is possibly a result of solvent effects and uncertainties in the experimental values, as each value was calculated from only a single reported experimental half-life and no full Eyring analysis was performed.

5. ^{1}H NMR Irradiation Studies

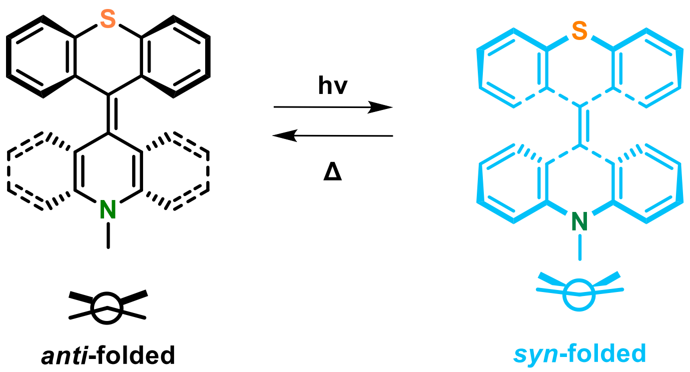

Scheme S1. Photochemical and thermal interconversion between **TX-Acr** in the *anti*- and *syn*-folded conformations.

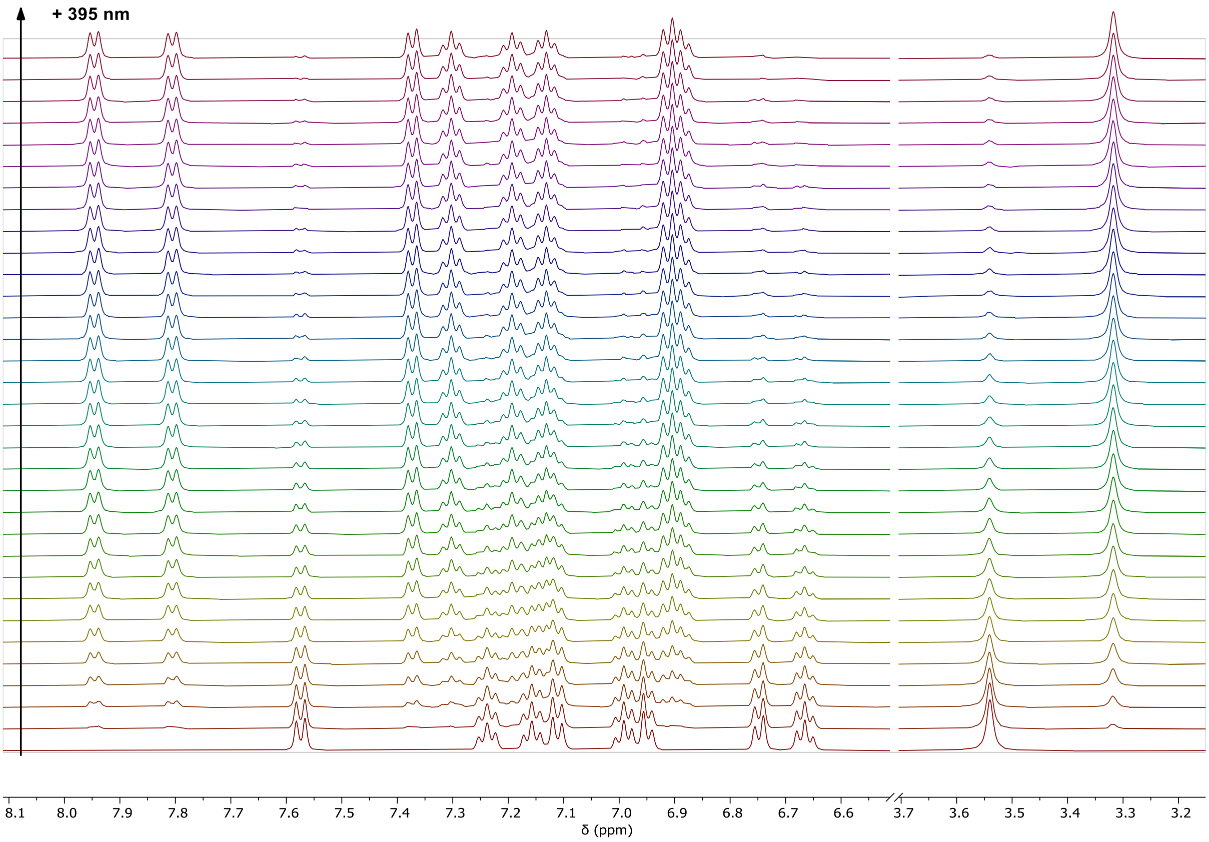

Figure S15. Stacked ^{1}H NMR spectra of **TX-Acr** in CD_2Cl_2 at ~–90 °C upon *in situ* irradiation with 395 nm light showing the conversion from the *anti*-folded conformation (bottom) to the *syn*-folded conformation (top).

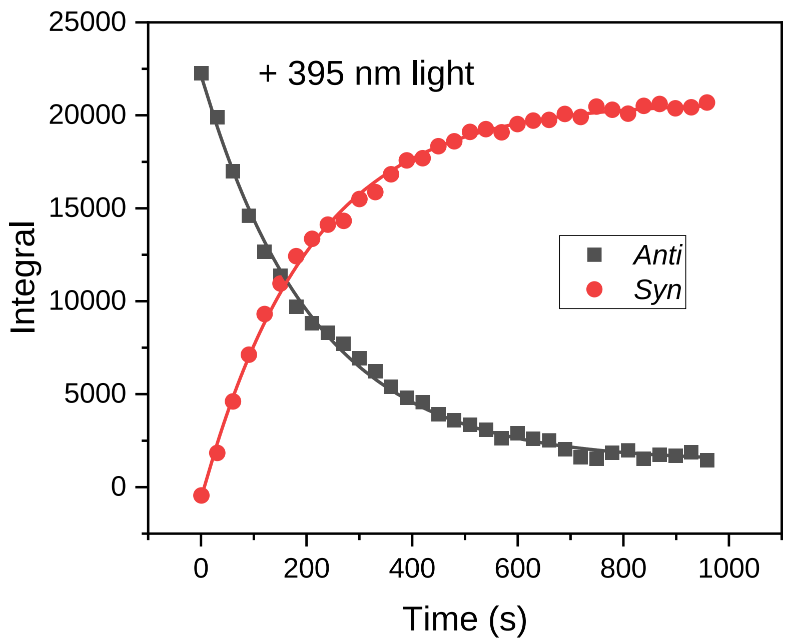

Figure S16. Photochemical interconversion of *anti*-folded to *syn*-folded **TX-Acr** in CD_2Cl_2 at ~–90 °C upon *in situ* irradiation with 395 nm light.

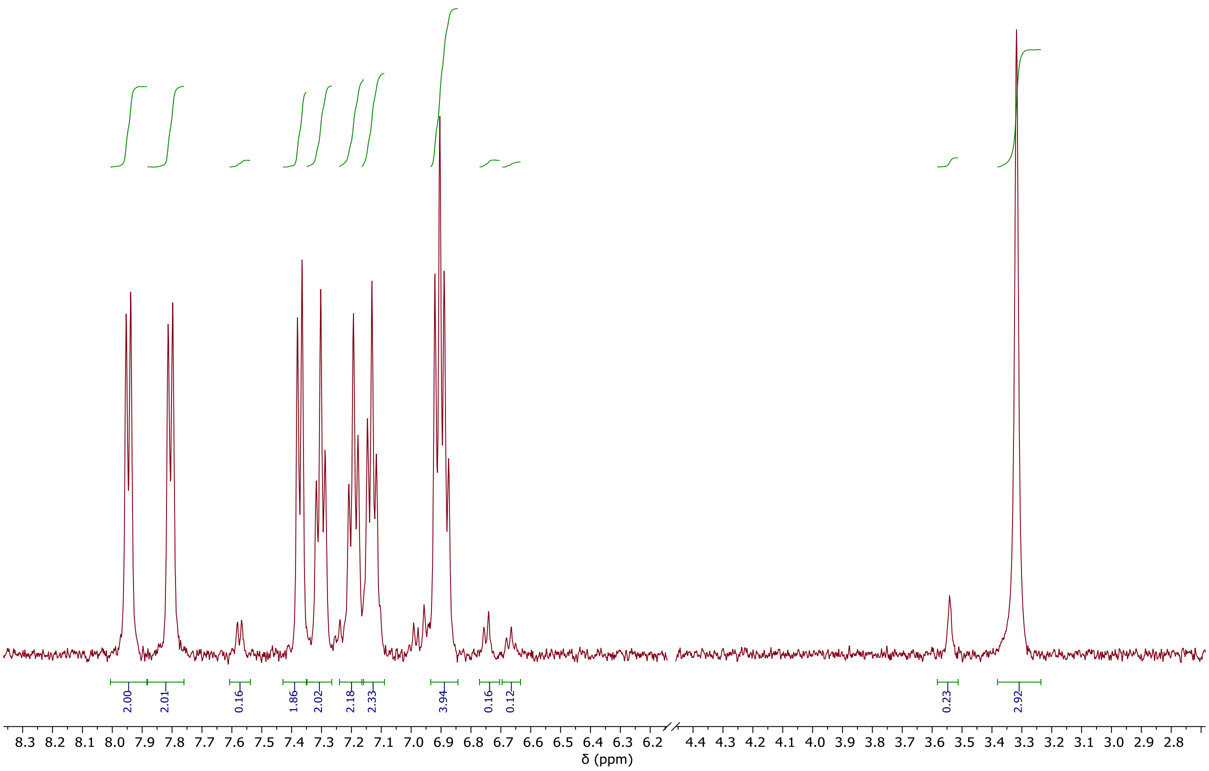

Figure S17. ^1H NMR spectrum of **TX-Acr** in CD_2Cl_2 at ~–90 °C upon *in situ* irradiation with 395 nm light at PSS showing almost quantitative conversion to the *syn*-folded state. The minor species corresponds to small amounts (<10%) of the *anti*-folded conformer.

As shown in Figure S18, the NOESY spectrum of the *in situ* generated **TX-Acr$_{syn}$** displays very strong through space contacts between the fjord protons on the opposite switch halves (blue arrows), which are even stronger than those to the adjacent aromatic protons (green arrows). This indicates that the distance between the former protons is closer which is consistent with a *syn*-folded conformation as shown in Scheme S1. This is also in good agreement with the computationally obtained structures of the *syn*-folded state (see Figure S11). In contrast, another potential symmetric photo-isomer, the twisted state, would have larger inter cleft proton distances and would thus display different NOE patterns.

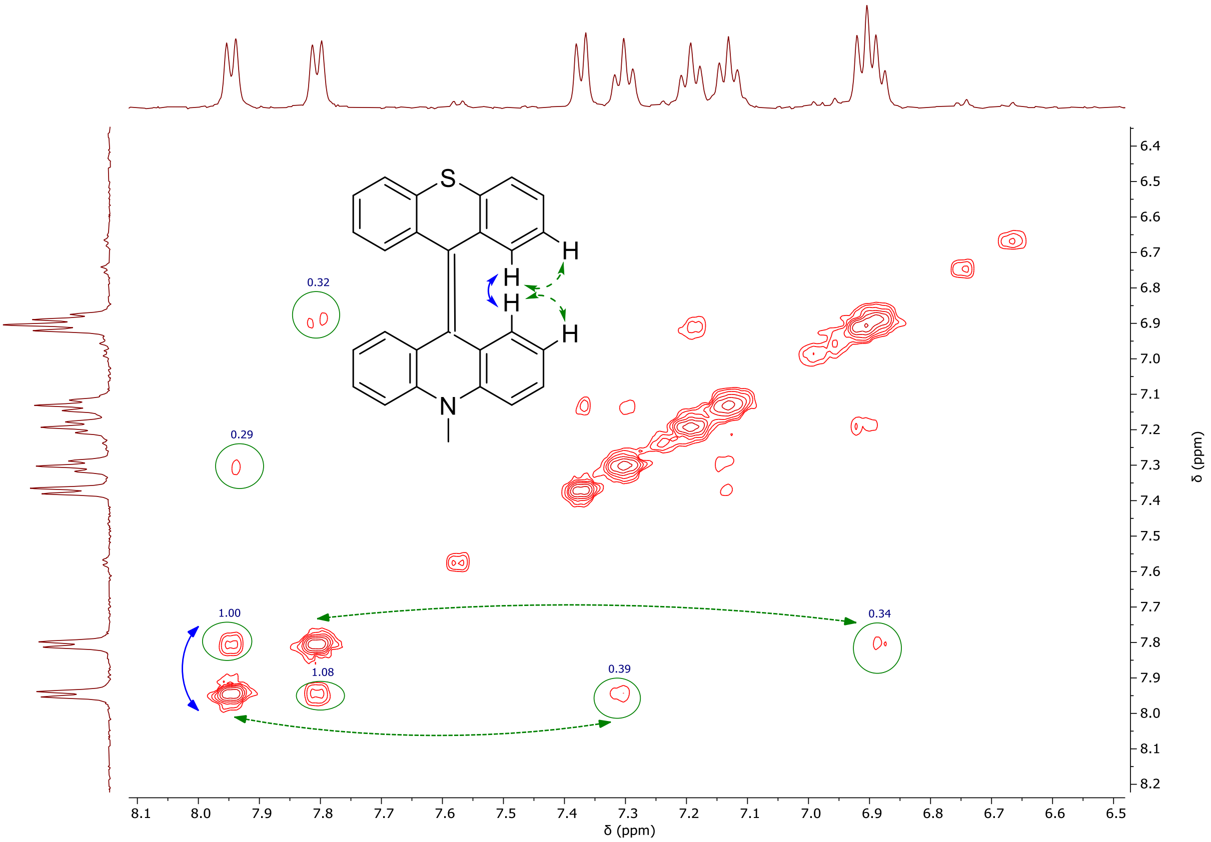

Figure S18. NOESY spectrum of *syn*-folded **TX-Acr** in CD_2Cl_2 at ~–90 °C upon *in situ* irradiation with 395 nm light. The blue arrows highlight the strongest NOE signal, arising from interactions of the cleft protons that point directly toward each other in the syn-folded conformation. Due to this close proximity this signal is stronger than that of the NOE contacts to the adjacent aromatic protons highlighted with green arrows.

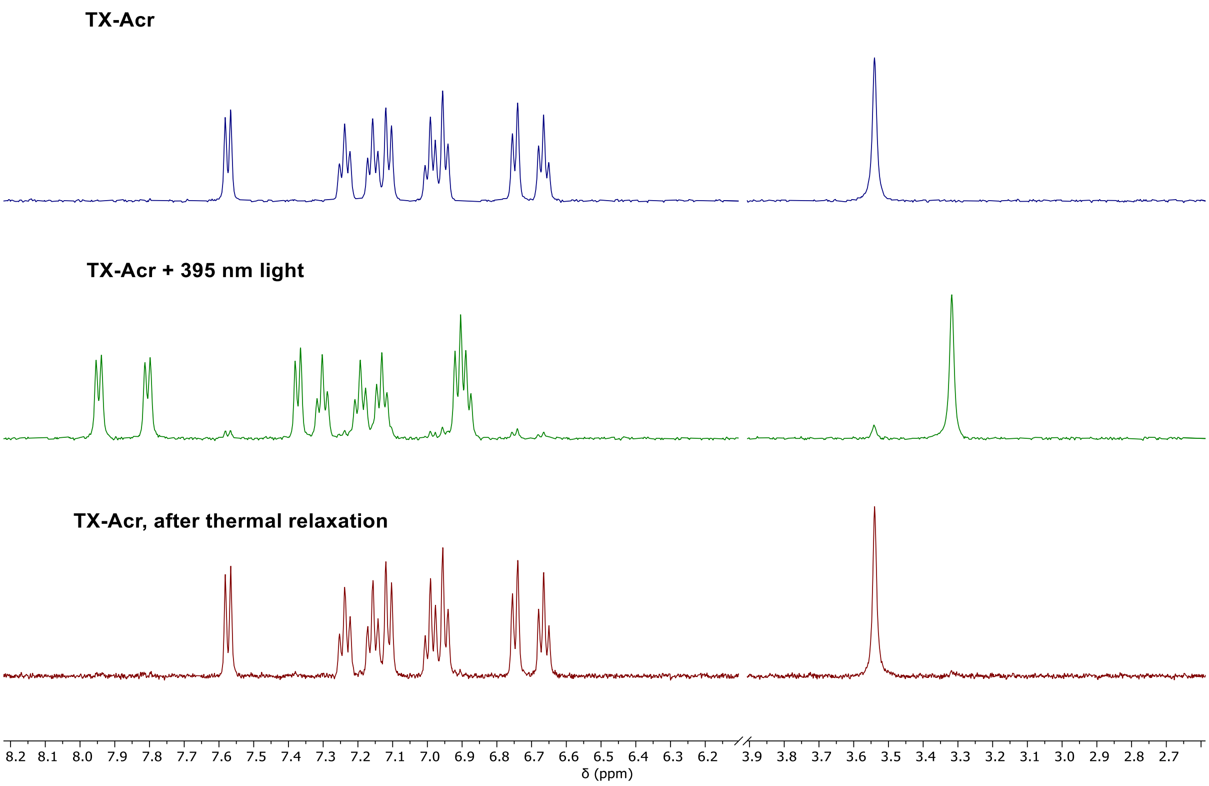

Figure S19. Stacked ^{1}H NMR spectra of **TX-Acr** in CD_2Cl_2 at ~–90 °C. Top: Native **TX-Acr** in the *anti*-folded state. Middle: Upon *in situ* irradiation with 395 nm light at PSS showing almost quantitative conversion to the *syn*-folded state. Bottom: After turning off the light and thermal relaxation back to the *anti*-folded state.

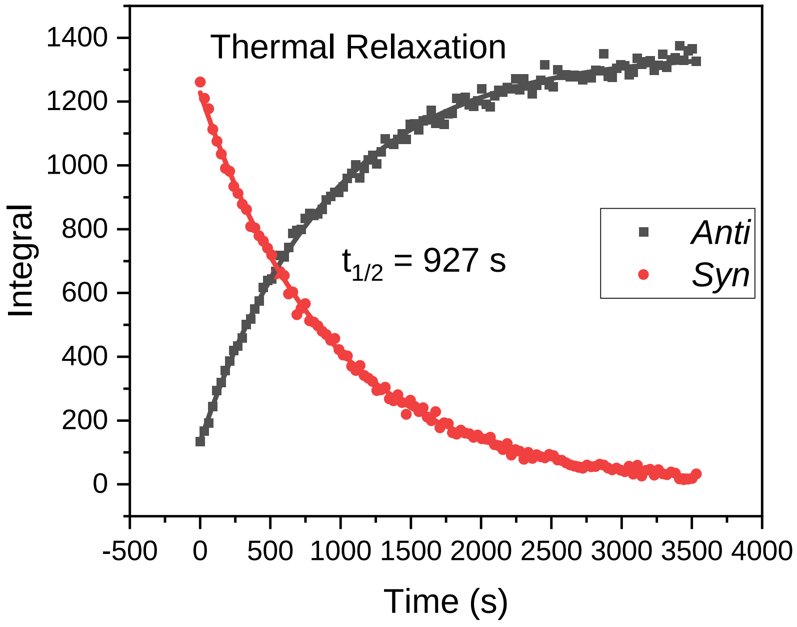

Figure S20. Thermal relaxation of *syn*-folded to *anti*-folded **TX-Acr** in CD_2Cl_2 at ~–90 °C. The half-life was determined by fitting to an exponential decay function. This corresponds to an energy barrier of ~55 kJ/mol; accordingly at room temperature the half-life would be ~0.5 ms.

6. Voltammetric Characterisation

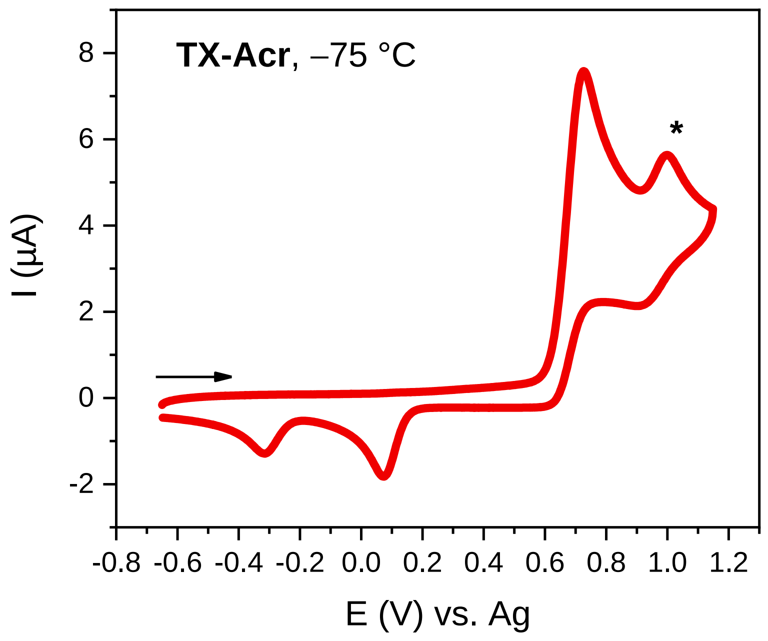

Figure S21. CV of **TX-Acr** in DCM, 100 mM $TBAPF_6$ at ca. –75 °C. The wave marked with an asterisk is only visible at these low temperatures and corresponds to the oxidation of **TX-Acr**$^{+\bullet}_{af}$ to **TX-Acr**$^{2+}_{af}$. A GC electrode was used as working electrode and a Ag wire was used as a pseudo-reference electrode.

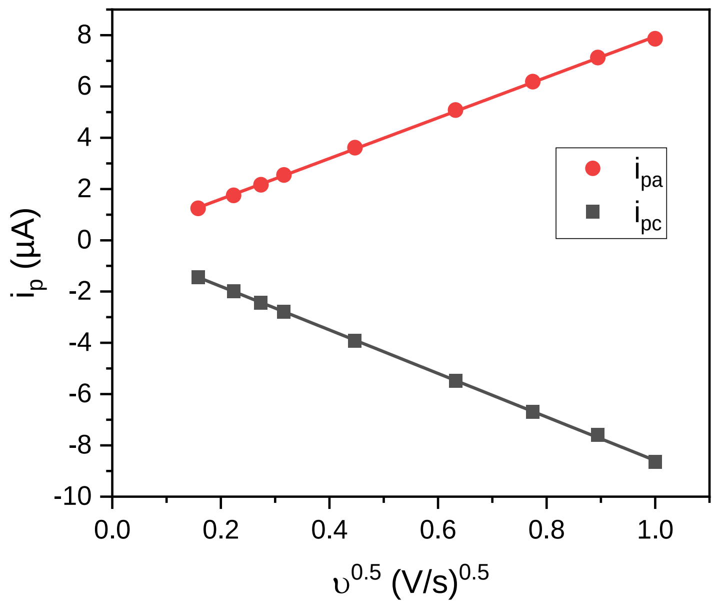

Figure S22. Anodic and cathodic peak currents of the reversible **TX-Acr**$^{2+}$**/ TX-Acr**$^{\bullet+}$ couple of 0.5 mM **TX-Acr**$^{2+}$ in CH_3CN, 100 mM $TBAPF_6$ as a function of the square root of the scan rate including linear fits. See Figure 7B for the corresponding CVs.

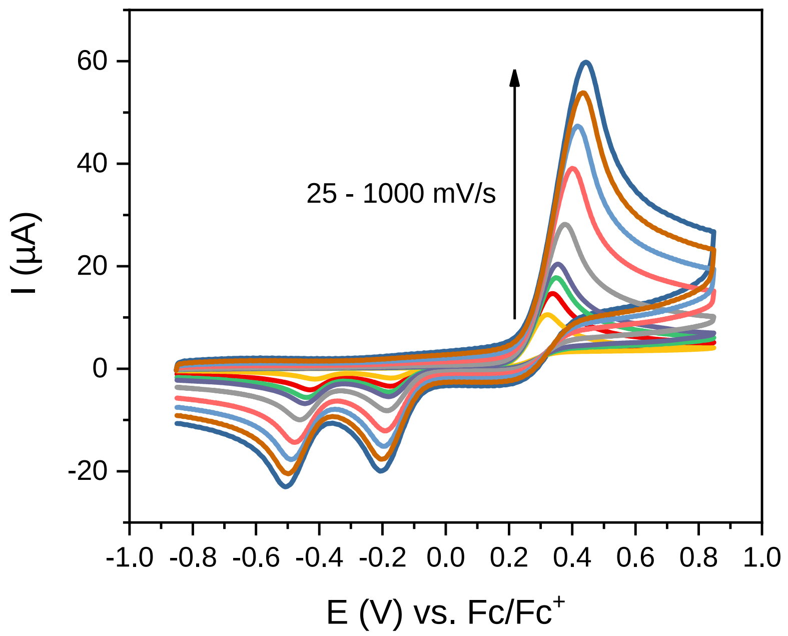

Figure S23. CVs of 0.5 mM **TX-Acr** in DCM, 100 mM $TBAPF_6$ at different scan rates.

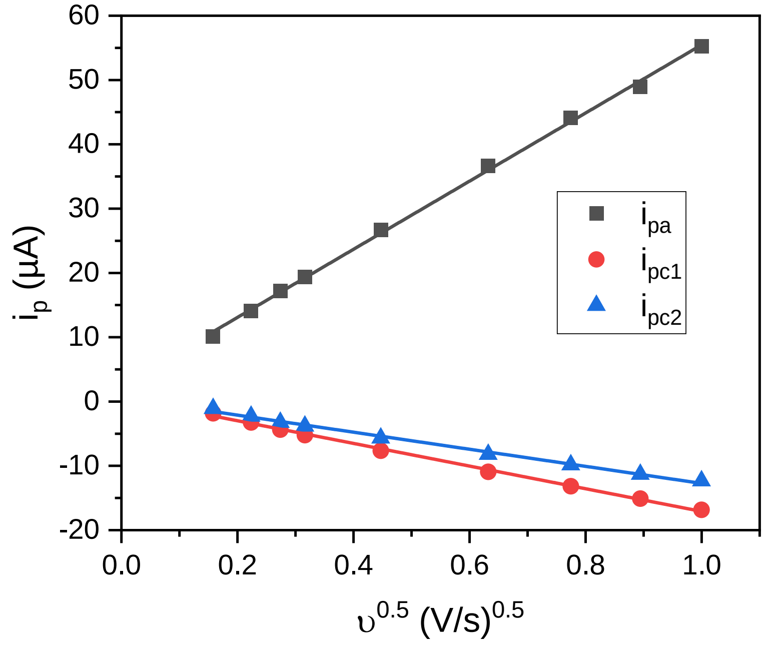

Figure S24. Anodic and cathodic peak currents of 0.5 mM **TX-Acr** in DCM, 100 mM $TBAPF_6$ as a function of the square root of the scan rate including linear fits.

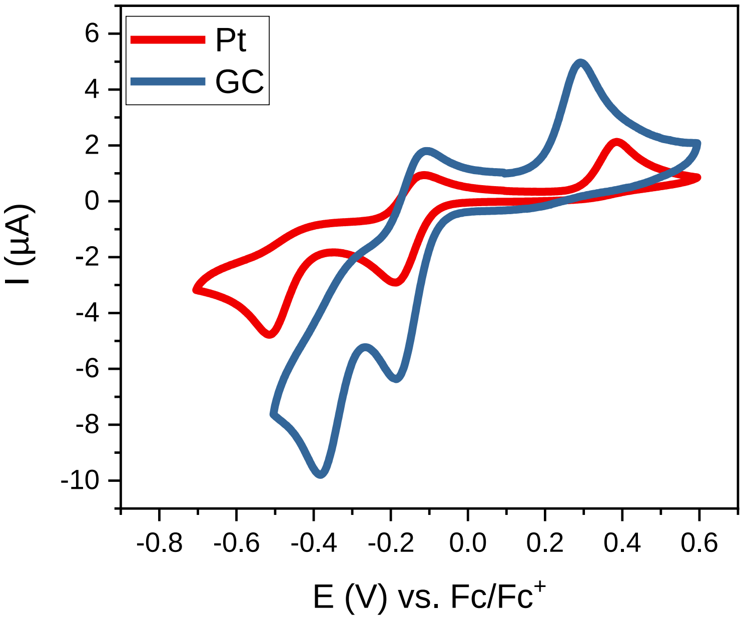

Figure S25. Comparison of CVs of 0.5 mM **TX-Acr^{2+}** in CH_3CN, 100 mM $TBAPF_6$ using either a Pt or GC working electrode.

7. Spectroelectrochemistry

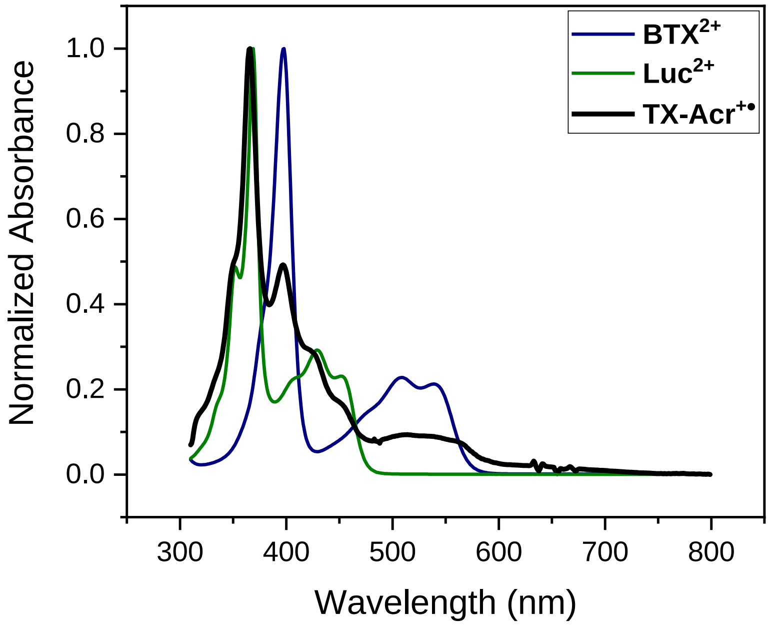

Figure S26. Comparison of the normalized UV-Vis spectra of **BTX^{2+}** and **Luc^{2+}** (in CH_3CN) with the spectroelectrochemically generated **TX-Acr$^{+\bullet}$** (in DCM/CH_3CN 1:1, 200 mM $TBAPF_6$).

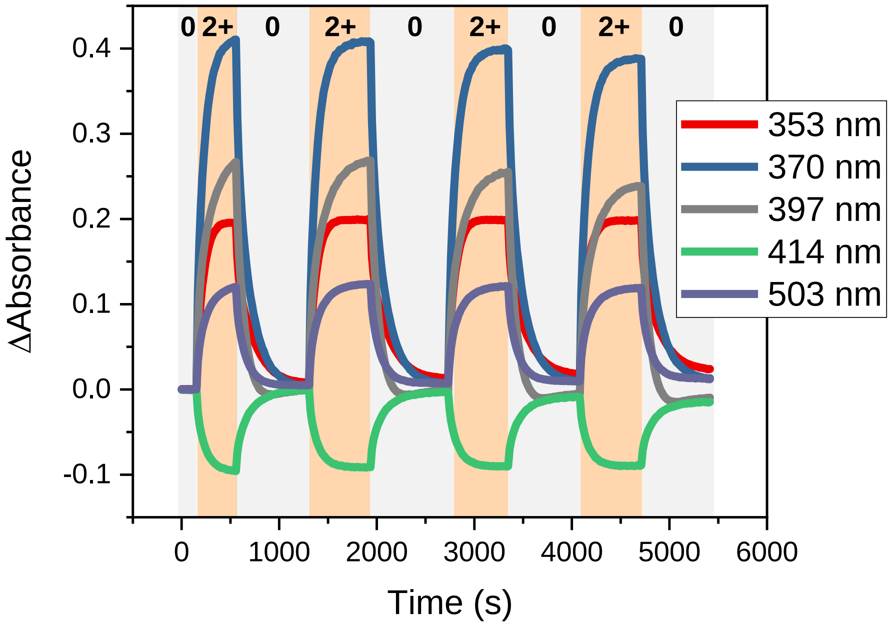

Figure S27. Changes in absorbance (difference spectrum) of **TX-Acr** in DCM/CH_3CN 1:1, 200 mM $TBAPF_6$ during repeat spectro-electrochemical cycling between the neutral and dicationic states.

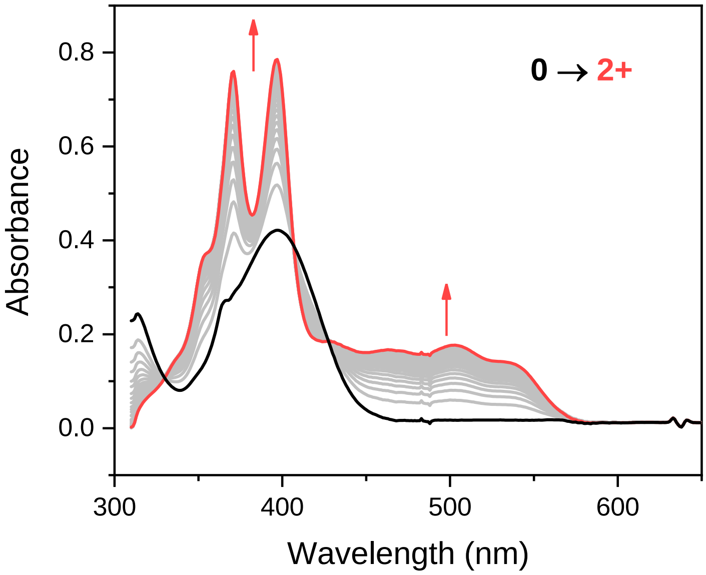

Figure S28. Selected UV-Vis spectra of spectroelectrochemical cycling of **TX-Acr** in DCM/CH_3CN 1:1, 200 mM $TBAPF_6$. Shown here is a cycle corresponding to the conversion from **TX-Acr** to **TX-Acr^{2+}** showing a clean, isosbestic conversion without intermediate states. The corresponding time-traces are shown in Figure 10.

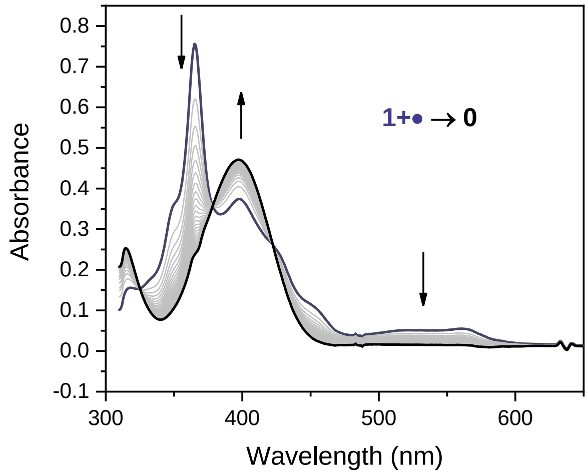

Figure S29. Selected UV-Vis spectra of spectroelectrochemical cycling of **TX-Acr** in DCM/CH_3CN 1:1, 200 mM $TBAPF_6$. Shown here is a cycle corresponding to the conversion from **TX-Acr**$^{+•}$ to **TX-Acr** showing a clean, isosbestic conversion without intermediate states. The corresponding time-traces are shown in Figure 10.

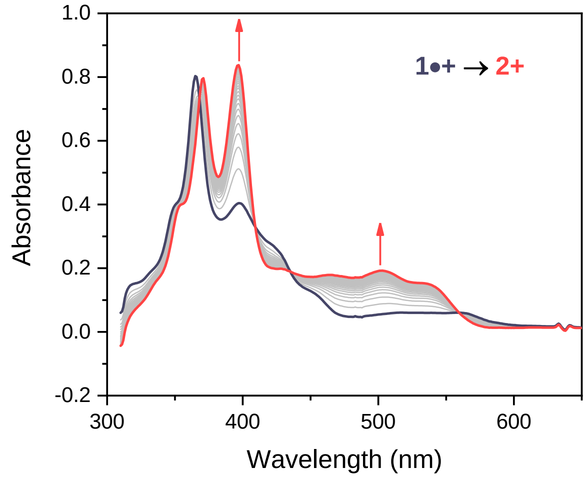

Figure S30. Selected UV-Vis spectra of spectroelectrochemical cycling of **TX-Acr** in DCM/CH_3CN 1:1, 200 mM $TBAPF_6$. Shown here is a cycle corresponding to the conversion from **TX-Acr**$^{+•}$ to **TX-Acr**$^{2+}$ showing a clean, isosbestic conversion without intermediate states. The corresponding time-traces are shown in Figure 10.

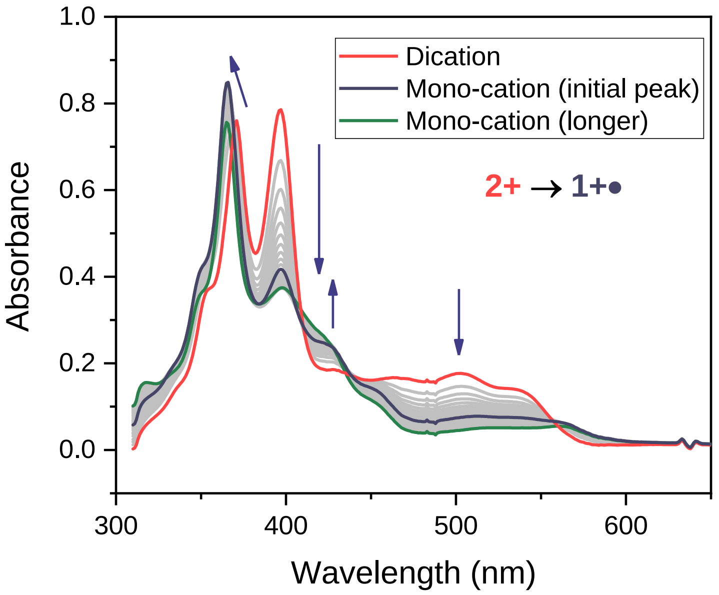

Figure S31. Selected UV-Vis spectra of spectroelectrochemical cycling of **TX-Acr** in DCM/CH_3CN 1:1, 200 mM $TBAPF_6$. Shown here is a cycle corresponding to the conversion from **TX-Acr^{2+}** to **TX-Acr$^{+•}$**. No clear isosbestic points were observed as upon prolonged reduction the initial spectrum of **TX-Acr$^{+•}$** (purple) slowly undergoes further changes (green) indicating a secondary process, which can be ascribed to the further reduction to the neutral **TX-Acr**, which slowly occurs at the applied potential of −0.32 V. The corresponding time-traces are shown in Figure 10.

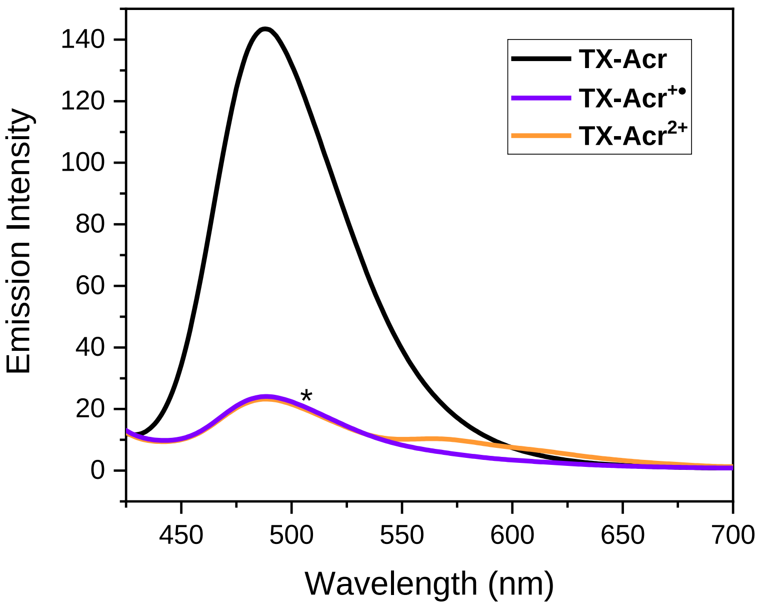

Figure S32. Fluorescence spectra of all three redox states of **TX-Acr** in DCM/CH_3CN 1:1, 200 mM $TBAPF_6$ generated spectroelectrochemically. The peak marked with an asterisk for the radical cation and the dication arises from the neutral state and is an artifact due to the measurement geometry. Neither of the cationic states shows emission in this region.

8. References

1. Corbet, B. P.; Wonink, M. B. S.; Feringa, B. L., Fast synthesis and redox switching of di- and tetra-substituted bisthioxanthylidene overcrowded alkenes. *Chem. Commun.* **2021,** *57* (62), 7665-7668.
2. Stopka, T.; Marzo, L.; Zurro, M.; Janich, S.; Wuerthwein, E. U.; Daniliuc, C. G.; Aleman, J.; Mancheno, O. G., Oxidative C-H Bond Functionalization and Ring Expansion with TMSCHN2: A Copper (I)-Catalyzed Approach to Dibenzoxepines and Dibenzoazepines. *Angew. Chem. Int. Ed.* **2015,** *54* (17), 5049-5053.
3. Papadopoulos, K.; Nikokavouras, J., Synthesis of N, N'-Dialkyl-9, 9'-Biacridylidenes and 9, 9'-Biacridinium Nitrates Containing Long Alkyl Chains. *Journal für Praktische Chemie/Chemiker-Zeitung* **1993,** *335* (7), 633-636.
4. Surgi, M. R.; Tirado-Rives, J., A convenient synthesis of 10, 10′-dimethyl-9, 9′-biacridylidene. *Org. Prep. Proced. Int.* **1988,** *20* (3), 295-298.
5. Krause, L.; Herbst-Irmer, R.; Sheldrick, G. M.; Stalke, D., Comparison of silver and molybdenum microfocus X-ray sources for single-crystal structure determination. *J. Appl. Cryst.* **2015,** *48* (1), 3-10.
6. Sheldrick, G. M., SHELXT–Integrated space-group and crystal-structure determination. *Acta Cryst. A* **2015,** *71* (1), 3-8.
7. Sheldrick, G. M., A short history of SHELX. *Acta Cryst. A* **2008,** *64* (1), 112-122.
8. Dolomanov, O. V.; Bourhis, L. J.; Gildea, R. J.; Howard, J. A.; Puschmann, H., OLEX2: a complete structure solution, refinement and analysis program. *J. Appl. Cryst.* **2009,** *42* (2), 339-341.
9. Neese, F.; Wennmohs, F.; Becker, U.; Riplinger, C., The ORCA quantum chemistry program package. *J. Chem. Phys.* **2020,** *152* (22).
10. Grimme, S.; Hansen, A.; Ehlert, S.; Mewes, J.-M., r2SCAN-3c: A “Swiss army knife” composite electronic-structure method. *J. Chem. Phys.* **2021,** *154* (6).
11. Grimme, S.; Brandenburg, J. G.; Bannwarth, C.; Hansen, A., Consistent structures and interactions by density functional theory with small atomic orbital basis sets. *J. Chem. Phys.* **2015,** *143* (5).
12. Bursch, M.; Mewes, J. M.; Hansen, A.; Grimme, S., Best-practice DFT protocols for basic molecular computational chemistry. *Angew. Chem. Int. Ed.* **2022,** *61* (42), e202205735.
13. Chai, J.-D.; Head-Gordon, M., Long-range corrected hybrid density functionals with damped atom–atom dispersion corrections. *Phys. Chem. Chem. Phys.* **2008,** *10* (44), 6615-6620.
14. Caldeweyher, E.; Ehlert, S.; Hansen, A.; Neugebauer, H.; Spicher, S.; Bannwarth, C.; Grimme, S., A generally applicable atomic-charge dependent London dispersion correction. *J. Chem. Phys.* **2019,** *150* (15).
15. Caldeweyher, E.; Bannwarth, C.; Grimme, S., Extension of the D3 dispersion coefficient model. *J. Chem. Phys.* **2017,** *147* (3).
16. Weigend, F.; Ahlrichs, R., Balanced basis sets of split valence, triple zeta valence and quadruple zeta valence quality for H to Rn: Design and assessment of accuracy. *Phys. Chem. Chem. Phys.* **2005,** *7* (18), 3297-3305.
17. Korenstein, R.; Muszkat, K. A.; Seger, G., Structure of the short wavelength photoisomers of bianthrone analogues. *J. Chem. Soc., Perkin Trans.* **1976,** (13), 1536-1540.

18. Korenstein, R.; Seger, G.; Muszkat, K. A.; Fischer, E., Reversible photochemistry of 10, 10′-dimethylbiacridan: internal and external heavy atom effects, and the structure of photoisomer F. *J. Chem. Soc., Perkin Trans.* **1977,** (5), 550-556.
19. Biedermann, P. U.; Stezowski, J. J.; Agranat, I., Conformational Space and Dynamic Stereochemistry of Overcrowded Homomerous Bistricyclic Aromatic Enes− A Theoretical Study. *Eur. J. Org. Chem.* **2001,** *2001* (1), 15-34.
